# Synthesis of a sucrose dimer with enone tether; a study on its functionalization

**DOI:** 10.3762/bjoc.10.124

**Published:** 2014-05-28

**Authors:** Zbigniew Pakulski, Norbert Gajda, Magdalena Jawiczuk, Jadwiga Frelek, Piotr Cmoch, Sławomir Jarosz

**Affiliations:** 1 Institute of Organic Chemistry, Polish Academy of Sciences, ul. Kasprzaka 44/52, 01-224 Warsaw, Poland

**Keywords:** CD-spectroscopy, Cotton effect, multivalent glycosystems, osmylation, stereoselective synthesis, sucrose

## Abstract

The reaction of appropriately functionalized sucrose phosphonate with sucrose aldehyde afforded a dimer composed of two sucrose units connected via their C6-positions (‘the glucose ends’). The carbonyl group in this product (enone) was stereoselectively reduced with zinc borohydride and the double bond (after protection of the allylic alcohol formed after reduction) was oxidized with osmium tetroxide to a diol. Absolute configurations of the allylic alcohol as well as the diol were determined by circular dichroism (CD) spectroscopy using the in situ dimolybdenum methodology.

## Introduction

Molecular recognition is one of the most important phenomena in stereoselective processes. Chiral crown ethers (or analogs) are particularly useful in enantioselective reactions [1–2] as well as differentiation of chiral guests [3–4]. From all of the chiral platforms designed for such receptors, sugars are the most promising due to their availability and biocompatibility. Up to date only monosaccharides have found a wide application in the synthesis of crown ether analogs [5–6]. The disaccharide scaffold is much less pronounced [7].

**Figure 1:**
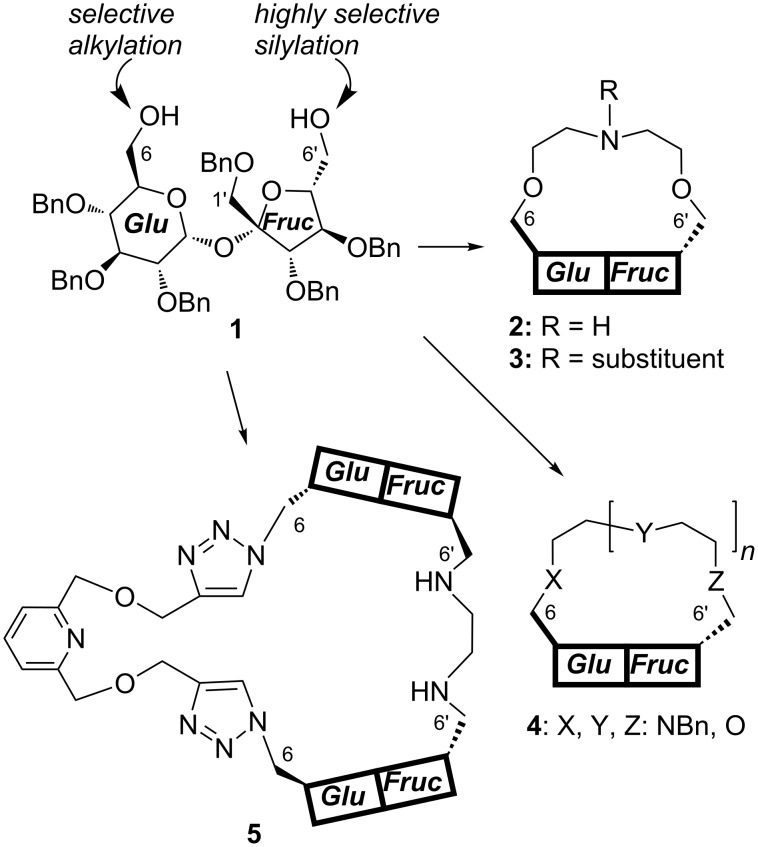
Examples of sucrose-based macrocycles.

During the past decade we have become engaged in the preparation of the analogs of crown and aza-crown ethers with sucrose scaffold. It is based on a selective protection of 1’,2,3,3’,4,4’-hexa-*O-*benzylsucrose (**1**) either at the glucose (C-6) [8] or fructose (C-6’) [9] end and further transformations to a variety of macrocycles (**2**–**4**; Figure 1). Such receptors exhibit interesting complexing properties towards chiral ammonium salts including amino acids [10–14]. More complex sucrose macrocycles, such as **5**, are available, although in rather low yield [15].

In this paper we present an approach to other derivatives containing two sucrose units. This type of dimers may be eventually used for the construction of macrocycles by (simple) connecting their C-6’ (fructose) ends.

## Results and Discussion

**Figure 2:**
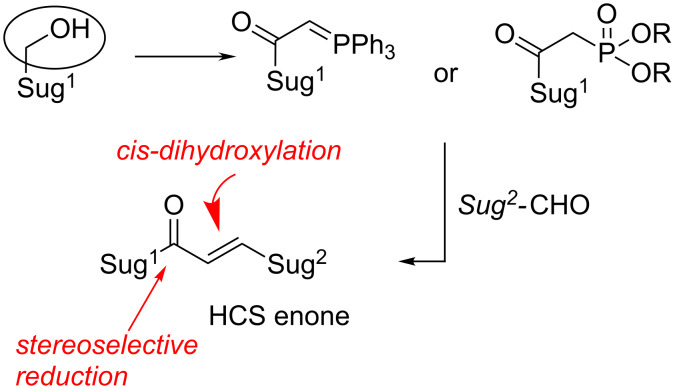
Synthesis of higher sugar precursors by a Wittig-type methodology.

Coupling of two sugar units can be performed by a number of methods. The best one in our hands was the Wittig-type methodology shown in Figure 2. The properly activated sugar is converted into phosphorane or phosphonate which – upon reaction with an aldehyde derived from another monosaccharide – provides higher carbon sugar (HCS) enone [16–18].

**Scheme 1:**
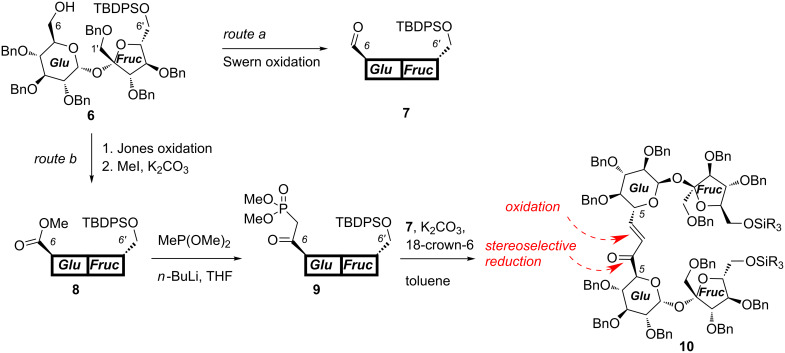
Synthesis of higher sugar enone **10**.

Application of this methodology to selectively protected 2,3,3’,4,4’-penta-*O-*benzylsucrose allowed us to elongate the parent disaccharide at either terminal position (1’,6’, and 6’) by relatively small (C2 or C7) unit and prepare so-called higher sucroses in good yields [19–20]. A similar approach is used now for a more convenient hexa-*O*-benzyl derivative which is easily silylated at the ‘fructose end’ providing alcohol **6** [8]. This alcohol was converted into aldehyde **7** [21] (route a in Scheme 1) and separately into phosphonate **9** (route b). Reaction of both synthons under the mild PTC conditions [22–24] afforded the respective enone **10** in good yield (Scheme 1).

Functionalization of the three-carbon atom unit connecting the C5-positions of both sucrose units required reduction of the carbonyl group of the enone system and oxidation of the double bond.

**Scheme 2:**
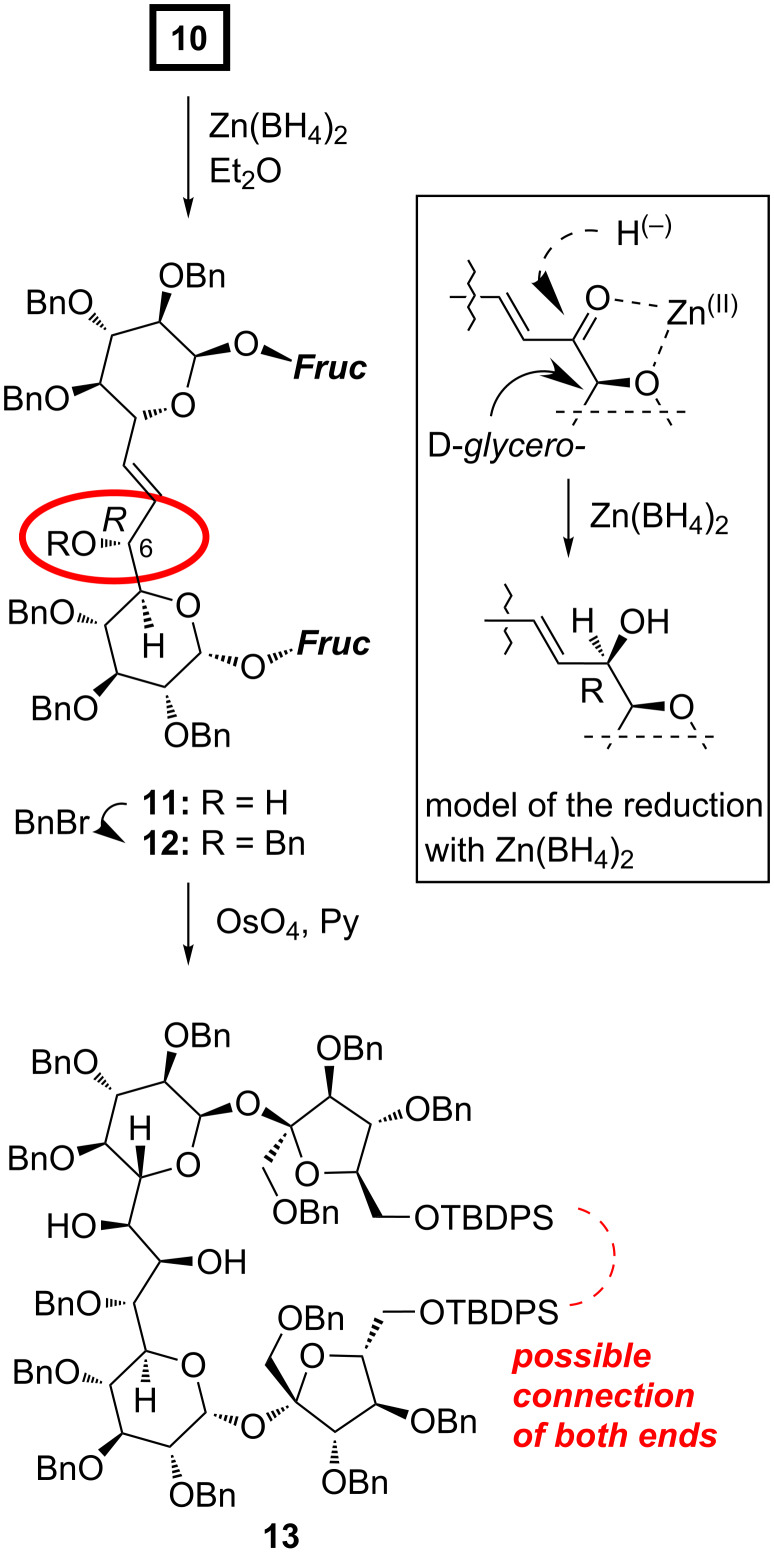
Synthesis of the diol **13** containing two sucrose units.

We have already reported that reduction of higher carbon sugar enones of the D series with zinc borohydride is highly selective and provides the corresponding allylic alcohols with the *R* configuration at the newly created stereogenic center, as the only products. This can be rationalized assuming the cyclic model of such reduction [25] shown in Scheme 2. We expected, therefore, also very high selectivity in the reduction of **10**.

Indeed, treatment of enone **10** with Zn(BH_4_)_2_ under the standard conditions afforded allylic alcohol **11** as single stereoisomer in 65% yield. Based on our model, the *R*-configuration might be safely assigned to the new stereogenic center. This assignment was further verified independently by circular dichroism spectroscopy (CD) using the in situ dimolybdenum methodology (see next chapter).

Next steps of the synthesis consisted of the protection of the C6–OH as benzyl ether (to **12**) and osmylation of the double bond. The *cis*-dihydroxylation provided, as single stereoisomer, a diol to which structure **13** could be assigned on the basis of the Kishi rule [26] (Scheme 2). It postulates that the attack of OsO_4_ occurs from the side opposite to hydroxy (alkoxy) substituent(s) flanking the double bond. Since in **12**, both alkoxy units act in the same direction, very high diastereoselectivity is not surprising. The assignment of the configuration of this diol was further confirmed also by the CD methodology; this is discussed in the next chapter.

### Determination of the absolute configuration of **11** and **13**

**Figure 3:**
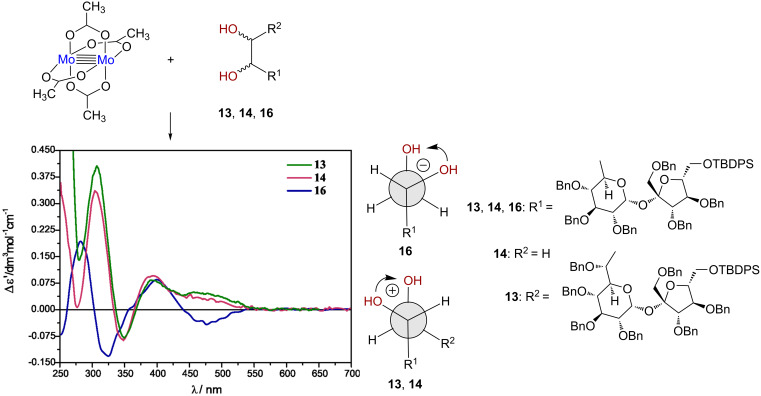
CD spectra of in situ formed chiral complexes of **13** (green line), **14** (purple line) and **16** (blue line) with dimolybdenum tetraacetate recorded in DMSO; right: preferred gauche conformation of the diols **13**, **14** and **16** in the complex with Mo_2_-core.

It is widely acceptable that the circular dichroism (CD) spectroscopy utilizing the in situ dimolybdenum methodology offers the hard proof of the absolute configuration of the *vic*-diols [27–29]. In this methodology, dimolybdenum tetraacetate acts as auxiliary chromophore allowing the application of electronic circular dichroism (ECD) to (otherwise in ECD non-observable) *vic*-diols. Mo_2_(OAc)_4_ when mixed with a chiral diol ligand forms complexes active in ECD in which a transfer of ligand chirality to the in situ-formed complex occurs in solution. Thus, stereochemistry of *vic*-diols can be easily assigned based on the helicity rule developed for this class of compounds. This rule correlates the positive/negative signs of Cotton effects (CE) occurring in the 300–400 nm spectral range in the ECD spectra with the positive/negative sign of the O–C–C–O torsion angle of the diol unit of resultant complexes with the Mo_2_-core. The basic assumption leading to the assignment of the absolute configuration (AC) based only on the ECD spectra with the Mo_2_-core preferring the gauche conformation of the diol units with both O–C–C–C fragments in an antiperiplanar arrangement (Figure 3). This arrangement is favored, for steric reasons, i.e., to avoid any interaction with the carboxylate ligands remaining in the stock complex. As a result of the structure–ECD spectra relationship, it is possible to assign the AC of the diol moiety unambiguously on the basis of the ECD spectra alone.

In the past few years, this simple but, above all, efficient and effective method is becoming more and more recognized as evidenced by the steadily increasing number of reports in the literature about its successful application in the determination of the AC of 1,2-diols [30–32].

Therefore, in assignment of the AC of compounds under the present study (**13**, **14** and **16**), we decided just to take advantage of the in situ methodology.

**Scheme 3:**
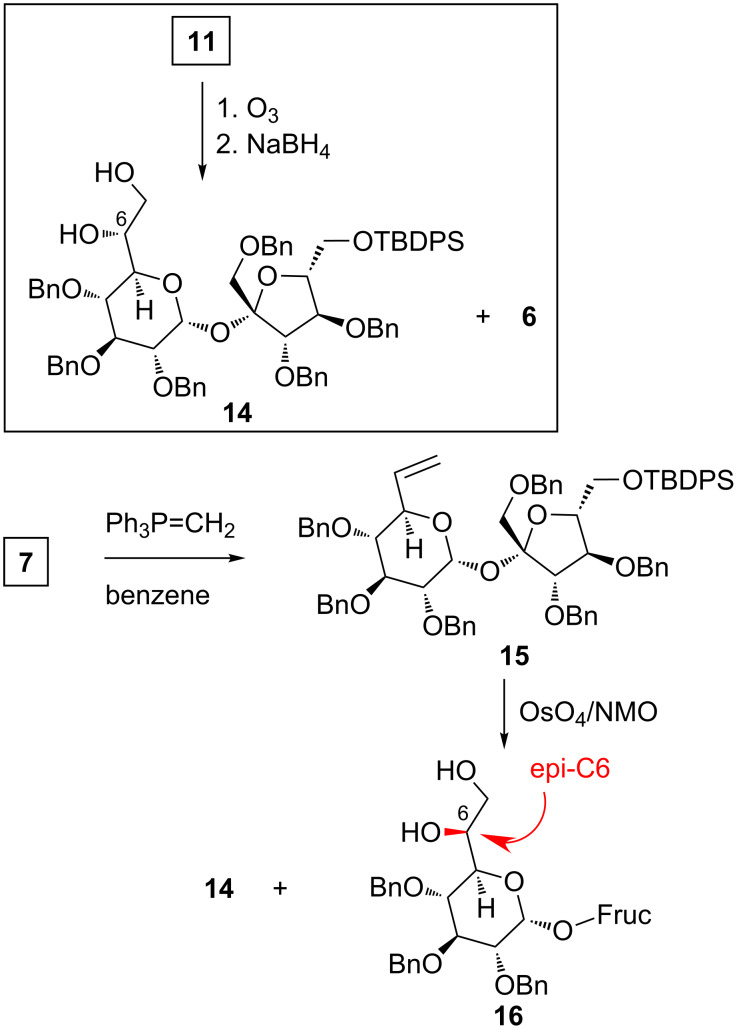
Synthesis of model sucrose diols.

This method was used to prove indirectly the 6*R* configuration at the newly created stereogenic center in allylic alcohol **11**. The double bond in **11** was cleaved with ozone and the resulting ozonide was reduced with NaBH_4_; this sequence afforded sucrose *vic*-diol **14** and (as a byproduct) sucrose alcohol **6** (Scheme 3).

The positive sign of the Cotton effect at around 307.0 nm recorded for the complex of the diol **14** with Mo_2_(OAc)_4_ unambiguously pointed at the 6*R* configuration (Figure 3). However, to exclude any errors we have also prepared **14** and epimeric alcohol **16**; its synthesis is shown in Scheme 3.

First, aldehyde **7** was converted into olefin **15** by treatment with the simplest Wittig reagent: Ph_3_P=CH_2_. Subsequent osmylation of the double bond in **15** provided two stereoisomeric diols in a 1:1 ratio; the first one was identical in all respects with the diol obtained from degradation of **11**.

The resultant ECD spectra of the Mo_2_-core with compounds **13** and **16** are shown in Figure 3. Based on the positive CE’s at 308.5 nm for **13** and negative at 310 nm for **16**, respectively, the positive (negative) sign of the O–C–C–O torsion angle has been attributed to these diols. In the next step, based on the preferred *gauche* conformation of the diol unit with both O–C–C–C fragments in an antiperiplanar arrangement as shown in Figure 3, we were able to assign unambiguously the (7*R*,8*R*) AC to diol **13** and (6*S*) to **16**.

## Conclusion

Coupling of two properly activated sucrose sub-units afforded the dimer in which both glucose-rings were connected via an enone linker. The dimer was then converted into a (partially protected) triol via a stereoselective reduction of the carbonyl group and highly selective *cis*-dihydroxylation of the double bond. The configuration at each new stereogenic center was determined by CD spectroscopy using the so-called dimolybdenum methodology which allows for fast, easy, and effective assignment of the absolute configuration of *vic*-diols. We have confirmed the usefulness of this simple methodology which can be applied even in cases when other spectroscopic methods fail.

It is worthy to point out that this methodology, which is used in the synthesis of more simple derivatives such as higher carbon sugars, was also applicable for the preparation of the sucrose dimer.

## Experimental

### General methods

All reported NMR spectra were recorded with a Varian-Vnmrs-600 MHz spectrometer (at 600 and 150 MHz for ^1^H and ^13^C NMR spectra, respectively) for solutions in CDCl_3_ at room temperature. Chemical shifts (δ, ppm) were determined relative to TMS as the internal standard. Most of the resonances were assigned by COSY (^1^H–^1^H) and gradient selected HSQC and HMBC correlations. Mass spectra were recorded with an ESI/MS Mariner (PerSeptive Biosystem) mass spectrometer. Elemental analyses were obtained using a Perkin-Elmer 2400 CHN analyzer. Optical rotations were measured with a Jasco P-2000 digital polarimeter for solutions in CHCl_3_ (*c* = 0.3) at room temperature. Flash and column chromatographic separations were performed on silica gel (Merck, 230–400 mesh). Progress of the reactions was monitored by thin-layer chromatography (TLC) performed on aluminum plates covered with silica gel (60 F254, Merck).

The ECD spectra were acquired at room temperature in DMSO (for UV-spectroscopy, Fluka) on a Jasco J-715 spectropolarimeter and were collected at 0.5 nm/step with an integration time of 0.25 s over the range 235–800 nm with 200 nm/min scan speed, 5 scans. For the ECD standard measurements the chiral diols (~3.6 mg, ca. 0.003 M) was mixed with stock complex [Mo_2_(O_2_CCH_3_)_4_] (Mo1) (~0.9 mg, ca. 0.002 M) and dissolved in DMSO (1 mL) so that the molar ratio of the stock complex to ligand was about 1:1.5 in general. Quantitative values could not be obtained with the in situ dimolybdenum method since the concentration of the chiral complex formed in solution and its actual structure were unknown. So the ECD data are given as the Δε' values, which are calculated in the usual manner by means of the equation Δε' = Δ*A*/*c* × *d*, *A* being the absorption, *c* the molar concentration of the chiral ligand, assuming 100% complexation, and *d* the path length of the cell.

The numbering of the atoms in sucrose dimers is as followed. The bottom part (green) is marked A and the top marked as B. The original numbering of the sucrose skeleton in both parts is retained (i.e., the glucose part is numbered C1–C6 and fructose C1'–C6').


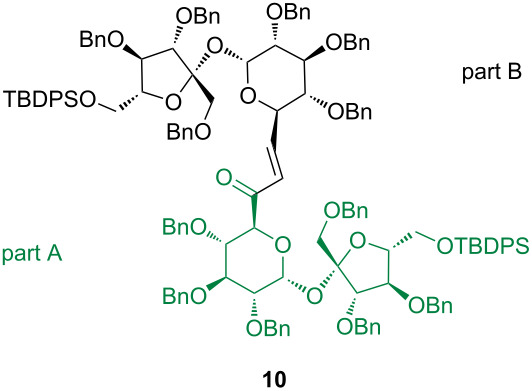


**Synthesis of phosphonate 9.** To a cooled −78 °C solution of dimethyl methylphosphonate (110 μL, 1.0 mmol) in THF (7 mL) a 2.5 M solution of butyllithium in hexane (0.4 mL, 1.0 mmol) was added and the mixture was stirred for 15 min. Then, a solution of **8** (325 mg, 0.28 mmol) in THF (5 mL) was slowly added and the mixture was stirred for additional 30 min. Reaction was quenched by addition of a saturated solution of NaCl (5 drops). The mixture was concentrated, and the residue was purified by column chromatography (hexane–ethyl acetate, 10:1 → 1:1) to afford title compound **9** (227 mg, 65%) as a foam. [α]_D_^20^ 16.2.; ^1^H NMR δ 7.64*–*7.61 (m, 4H, Ar), 7.35*–*7.18 (m, 36H, aryl-H), 6.08 (d, 1H, *J*_1,2_ = 3.6 Hz, H-1), 4.86–4.38 (m, 12H, PhC*H_2_*), 4.54 (d, 1H, *J*_5,4_ = 9.9 Hz, H-5), 4.48 (d, 1H, *J*_3,4_ = 7.5 Hz, H-3'), 4.45 (dd, 1H, *J*_4,3_ = 7.5, *J*_4,5_ = 15.0 Hz, H-4'), 4.03 (dd, 1H, *J*_6,5_ = 3.8, *J*_6,6’_ 11.5 Hz, H-6'), 3.94 (m, 2H, H-3, H-5'), 3.85 (dd, 1H, *J*_6,5_ = 4.1 Hz, *J*_6,6’_ = 11.5 Hz, H-6'), 3.71 (dd, 1H, *J*_4,3_ = 9.1 Hz, *J*_4,5_ = 9.9 Hz, H-4), 3.67 (d, 1H, *J*_gem_ = 10.8 Hz, H-1'), 3.62 (d, 3H, *J*_H,P_ = 11.1 Hz, OCH_3_), 3.61 (d, 3H, *J*_H,P_ = 11.2 Hz, OCH_3_), 3.56 (d, 1H, *J*_gem_ = 10.8 Hz, H-1'), 3.43 (dd, 1H, *J*_2,1_ = 3.6, *J*_2,3_ = 9.7 Hz, H-2), 3.26 (dd, 1H, *J*_7,7'_ = 15.7 Hz, *J*_H,P_ = 20.3 Hz, H-7), 2.97 (dd, 1H, *J*_7,7'_ = 15.7 Hz, *J*_H,P_ = 20.8 Hz, H-7), 1.06 (s, 9H, *t-*Bu); ^13^C NMR δ 198.5 (d, *J* 7.4 Hz, C=O), 138.7, 138.5, 138.2, 138.1, 137.8, 137.7, 135.6, 135.4, 133.1, 132.7, 129.8, 129.8, 128.6–127.5 (Ar), 104.5 (C-2'), 89.2 (C-1), 83.3 (C-3'), 81.5 (C-3), 80.8 (C-4'), 80.5 (C-5'), 79.5 (C-2), 77.9 (C-4), 75.7 (Ph*C*H_2_), 74.9 (d, *J* 5.0 Hz, C-5), 74.8 (Ph*C*H_2_), 73.5 (Ph*C*H_2_), 73.4 (Ph*C*H_2_), 72.9 (Ph*C*H_2_), 72.0 (Ph*C*H_2_), 72.0 (C-1'), 63.5 (C-6'), 52.8 (d, *J* = 6.3 Hz, OCH_3_), 52.6 (d, *J* = 6.4 Hz, OCH_3_), 37.9 (d, *J* = 136.9 Hz, C-7), 26.9 (*t-*Bu-CH_3_), 19.2 (*t-*Bu-C); ^31^P NMR (CDCl_3_) δ 23.5; anal. calcd for C_73_H_81_O_14_PSi (1241.51): C, 70.62; H, 6.58; found: C, 70.44; H, 6.79.

**Synthesis of ketone 10.** To a solution of aldehyde **7** (200 mg, 0.18 mmol), phosphonate **9** (220 mg, 0.18 mmol), and 18-crown-6 (70 mg) in toluene (25 mL) potassium carbonate (300 mg) was added and the suspension was stirred at rt for 4 days. The solvents were evaporated and the residue was purified by column chromatography (hexane–ethyl acetate, 40:1 → 5:1) to afford title compound **10** as a foam (287 mg; 73%). [α]_D_^20^ 41.8; ^1^H NMR δ 7.62*–*7.61 (m, 8H, Ar), 7.28*–*7.06 (m, 73H, Ar, =*C*H-CO), 6.75 (dd, 1H, *J* = 1.8 Hz, 15.7 Hz, =C*H*), 5.90 (d, 1H, *J* = 3.5 Hz, H-1A), 5.82 (d, 1H, *J* = 3.5 Hz, H-1B), 4.74*–*4.26 (m, 24H, 12 × PhC*H_2_*), 4.67 (d, 1H, *J* = 10.2 Hz, H-5A), 4.58 (d, 1H, *J* = 12.3 Hz, H-5B), 4.37*–*4.26 (m, 4H, furanose), 4.00*–*3.80 (m, 8H, H-3A, H-3B, 4 × H-6', 2 × furanose), 3.77 (d, 1H, *J* = 11.0 Hz, H-1'B), 3.68 (d, 1H, *J* = 10.9 Hz, H-1'A), 3.50 (dd, 1H, *J* = 9.1 Hz, 10.0 Hz, H-4A), 3.46 (d, 1H, *J* = 10.9 Hz, H-1'A), 3.42*–*3.38 (m, 2H, H-1'B, H-2A), 3.25 (dd, 1H, *J* = 3.5 Hz, 9.6 Hz, H-2A), 2.97 (dd, 1H, *J* = 9.1 Hz, 9.9 Hz, H-4B), 1.03 (s, 9H, *t-*Bu), 1.02 (s, 9H, *t-*Bu); ^13^C NMR δ 195.2 (C=O), 144.7 (=*C*H-CO), 138.9, 138.8, 138.4, 138.3, 138.2, 138.1, 138.1, 138.0, 137.9, 137.9, 137.8, 137.7, 135.6, 135.6, 135.5, 133.4, 133.3, 133.2, 133.1, 129.7, 129.7, 129.7, 129.6, 128.4–127.4 (Ar), 124.8 (=*C*H) 105.1 (C-2'A), 104.8 (C-2'B), 90.3 (C-1A), 90.2 (C-1B), 84.4, 83.9, 83.3, 83.2, 81.8 (C-4B), 81.7, 81.7, 81.5 (C-3A), 81.4 (C-3B), 79.8 (C-2A, C-2B), 79.6 (C-4A), 75.7 (Ph*C*H_2_), 75.4 (Ph*C*H_2_), 74.9 (C-5A, Ph*C*H_2_), 74.4 (Ph*C*H_2_), 73.5 (Ph*C*H_2_), 73.4 (Ph*C*H_2_), 73.1 (Ph*C*H_2_), 73.0 (Ph*C*H_2_), 72.6 (Ph*C*H_2_), 72.4 (Ph*C*H_2_), 72.1 (Ph*C*H_2_),71.8 (Ph*C*H_2_), 70.6 (C-1'), 70.4 (C-1'), 69.8 (C-5B), 65.3 (C-6'), 65.0 (C-6'), 27.0 (*t-*Bu), 27.0 (*t-*Bu), 19.3 (CH_3_), 19.3 (CH_3_); anal. calcd for C_141_H_148_O_21_Si_2_ (2234.91): C, 75.78; H, 6.68; found: C, 75.59; H, 6.80.

**Stereoselective reduction of ketone 10.** To an ice-cooled solution of ketone **10** (270 mg, 0.12 mmol) in Et_2_O (15 mL), an etheral solution of zinc borohydride (0.6 mmol) was added and the mixture was stirred at 0 °C for 1 h. Water (10 drops) was added to decompose excess of hydride, the solvents were evaporated, and the residue was purified by column chromatography (hexane–ethyl acetate, 10:1 → 5:1) to afford title compound **11** (176 mg, 65%) as a foam. [α]_D_^20^ 40.4; ^1^H NMR δ 7.63*–*7.61 (m, 8H, Ar), 7.30*–*7.05 (m, 72H, Ar), 5.97 (dd, 1H, *J* = 7.9 Hz, 15.6 Hz, =C*H*-CHOH), 5.83 (d, 2H, *J* = 3.6 Hz, H-1A, H-1B), 5.76 (dd, 1H, *J* = 5.5 Hz, 15.6 Hz, =C*H*), 4.80*–*4.73 (m, 5H, PhC*H_2_*), 4.63*–*4.41 (m, 17H, PhC*H*_2_, H-3', H-5B), 4.39*–*4.34 (m, 6H, PhC*H*_2_, H-3', H-3', H-4', H-6A), 4.32*–*4.28 (m, 1H, H-4'), 4.25 (dd, 1H, *J* = 2.2 Hz, 10.3 Hz, H-5A), 4.13 (d, 1H, *J* = 10.9 Hz, PhC*H*_2_), 4.06*–*4.04 (m, 1H, H-5'), 3.99*–*3.95 (m, 2H, 2 × H-6'), 3.92*–*3.89 (m, 2H, H-5', H-6'), 3.86*–*3.79 (m, 3H, H-3A, H-1', H-3B), 3.65 (d, 1H, *J* = 11.0 Hz, H-1'), 3.52 (d, 1H, *J* = 11.0 Hz, H-1'), 3.49 (d, 1H, H-1'), 3.35 (dd, 1H, *J* = 3.5 Hz, 9.6 Hz, H-2B), 3.27*–*3.24 (m, 1H, H-4A), 3.21*–*3.16 (m, 2H, H-2A, H-4B), 1.03 (s, 9H, *t-*Bu), 1.02 (s, 9H, *t-*Bu); ^13^C NMR δ 139.0, 138.9, 138.6, 138.6, 138.5, 138.4, 138.3, 138.1, 138.0, 137.9, 137.8, 137.7, 135.7, 135.6, 135.5, 135.5, 133.5, 133.3, 133.3, 132.8, 131.3 (=*C*H), 130.4 (=*C*H-CHOH), 129.8, 129.7, 129.7, 129.6, 128.5–127.3 (Ar), 105.0 (C-2'), 104.5 (C-2'), 90.2 (C-1B), 89.0 (C-1A), 84.5 (C-3'), 83.6 (C-4'), 83.1 (C-4'), 82.3 (C-3A), 82.2 (C-4B), 81.8 (C-5'), 81.6 (C-3'), 81.5 (C-3B), 81.0 (C-5'), 80.4 (C-2A), 80.0 (C-2B), 78.4 (C-4A), 75.5 (Ph*C*H_2_), 75.4 (Ph*C*H_2_), 74.6 (Ph*C*H_2_), 74.4 (Ph*C*H_2_), 73.6 (Ph*C*H_2_), 73.4 (Ph*C*H_2_), 73.4 (C-5A), 73.1 (Ph*C*H_2_), 73.0 (Ph*C*H_2_), 72.7 (Ph*C*H_2_), 72.6 (Ph*C*H_2_), 71.8 (Ph*C*H_2_), 71.7 (Ph*C*H_2_), 71.5 (C-6A), 71.4 (C-1'), 70.5 (C-1'), 70.0 (C-5B), 65.8 (C-6'), 63.7 (C-6'), 27.0 (*t-*Bu), 27.0 (*t-*Bu), 19.3 (CH_3_), 19.3 (CH_3_); anal. calcd for C_141_H_150_O_21_Si_2_ (2236.92): C, 75.71; H, 6.76; found: C, 75.51; H, 6.63.

**Benzylation of 11.** To a solution of alcohol **11** (82 mg, 0.037 mmol) in DMF (1 mL), sodium hydride (60% suspension in mineral oil, 7 mg) was added and the mixture was stirred for 30 min at rt. Benzyl bromide (11 μL, 0.092 mmol) was added, and stirring was continued overnight. Excess of sodium hydride was decomposed with methanol (0.5 mL). The product was isolated by column chromatography (hexane–ethyl acetate, 10:1 → 5:1) to afford **12** (34 mg, 40%) as a foam. [α]_D_^20^ 21.8 (*c* 0.9, CH_2_Cl_2_); ^1^H NMR δ 7.67*–*7.63 (m, 8H, Ar), 7.28*–*7.00 (m, 77H, Ar), 5.93*–*5.89 (m, 2H, H-1B, =C*H*-CHOH), 5.63*–*5.59 (m, 2H, H-1A, =C*H*), 4.79*–*4.50 (m, 13H, H-5B, PhC*H_2_*), 4.47*–*4.16 (m, 18H, H-5A, PhC*H*_2_), 4.10*–*3.75 (m, 12H, H-6A, 4 × H-6', H-3B, 2 × H-1', H-3A, 2 × furanose, PhC*H*), 3.56*–*3.52 (m, 2H, 2 × H-1'), 3.41 (dd, 1H, *J* = 3.6, 9.7 Hz, H-2B), 3.19*–*3.14 (m, 2H, H-4A, H-4B), 2.92 (dd, 1H, *J* = 3.5, 9.6 Hz, H-2A), 1.03 (s, 9H, *t-*Bu), 1.03 (s, 9H, *t-*Bu); ^13^C NMR δ: 139.0, 138.8, 138.7, 138.6, 138.5, 138.4, 138.3, 138.3, 138.3, 138.3, 138.2, 138.0, 137.8, 135.6, 135.5, 135.5, 135.5, 134.4 (=*C*H), 133.7, 133.6, 133.4, 133.2, 129.7, 129.6, 129.6, 129.6, 128.3–127.0 (Ar), 104.4 (C-2'), 104.4 (C-2'), 90.1 (C-1A), 89.9 (C-1B), 84.5, 84.1, 83.2, 82.2, 82.0 (C-3A), 82.0 (C-4B), 81.6 (C-3B, C-6A), 80.4 (C-2A), 79.9 (C-2B), 78.6, 78.2 (C-4A), 75.6 (Ph*C*H_2_), 75.2 (Ph*C*H_2_), 74.6 (Ph*C*H_2_), 74.2 (Ph*C*H_2_), 73.6 (Ph*C*H_2_), 73.2 (Ph*C*H_2_), 73.1 (Ph*C*H_2_), 73.0 (Ph*C*H_2_), 72.7 (Ph*C*H_2_), 72.6 (C-5A), 72.5 (Ph*C*H_2_), 71.9 (Ph*C*H_2_), 71.8 (Ph*C*H_2_), 71.0 (C-1'), 70.3, 70.2 (C-1'), 69.9, 66.7 (C-6'), 65.6 (C-6'), 27.0 (*t-*Bu), 26.9 (*t-*Bu), 19.3 (CH_3_), 19.3 (CH_3_); anal. calcd for C_148_H_156_O_21_Si_2_ (2327.05): C, 76.39; H, 6.76; found: C, 76.44; H, 6.73.

**Dihydroxylation of the double bond of 12.** Olefin **12** (60 mg, 0.026 mmol) and OsO_4_ (30 mg, 0.120 mmol) were dissolved in pyridine (4 mL), and stirred for 48 h. The solvent was evaporated, the residue was dissolved in ethyl acetate (10 mL), to which sat. aq Na_2_S_2_O_3_ (1 mL) was added, and the suspension was stirred for 2 days. The organic layer was separated, solvents were evaporated to dryness and the residue was purified by column chromatography (hexane–ethyl acetate, 5:1) to afford **13** (44 mg, 73%) as colorless glass. [α]_D_^20^ 22.8; ^1^H NMR δ 7.64*–*7.59 (m, 8H, Ar), 7.27*–*6.98 (m, 77H, Ar), 5.83 (bs, 1H, H-1A), 5.80 (d, 1H, *J*_1,2_ = 3.5 Hz, H-1B), 4.82*–*4.69 (m, 5H, PhC*H_2_*), 4.65*–*4.49 (m, 11H, PhC*H*_2_, sugar-H), 4.45*–*4.34 (m, 10H, PhC*H*_2_, 2 × sugar-H), 4.29*–*4.23 (m, 7H, PhC*H*_2_, 5 × sugar-H), 4.08*–*4.05 (m, 2H, PhC*H*_2_, sugar-H), 4.01*–*3.95 (m, 6H, 3 × H-6', 3 × sugar-H), 3.89*–*3.78 (m, 4H, H-1', H-3A, H-3B, H-6'), 3.67 (d, 1H, *J* = 11.1 Hz, H-1'), 3.58*–*3.55 (m, 2H, H-1', H-1'), 3.37 (dd, 1H, *J* = 3.6 Hz, 9.6 Hz, H-2B), 3.36*–*3.32 (m, 2H, H-2A, sugar-H), 1.02 (s, 9H, *t-*Bu), 1.01 (s, 9H, *t-*Bu); ^13^C NMR δ 139.1, 139.0, 138.9, 138.8, 138.6, 138.3, 138.2, 138.2, 138.0, 137.9, 137.7, 137.6, 135.6, 135.6, 135.5, 135.5, 133.6, 133.4, 133.3, 132.9, 129.7, 129.7, 129.6, 129.5, 128.4-127.1 (Ar), 105.1 (C-2'), 104.7 (C-2'), 90.7 (C-1B), 89.4 (C-1A), 84.4, 83.5, 82.5 (C-3B), 82.0, 81.9, 81.6 (C-3A), 81.2, 80.4 (C-2B), 79.7 (C-2A), 79.3, 78.6, 75.5 (Ph*C*H_2_), 75.3 (Ph*C*H_2_), 74.6 (Ph*C*H_2_), 74.3, 74.3 (Ph*C*H_2_), 73.5 (Ph*C*H_2_), 73.2 (Ph*C*H_2_), 73.1 (Ph*C*H_2_), 73.1 (Ph*C*H_2_), 72.7 (Ph*C*H_2_), 72.5 (Ph*C*H_2_), 71.9 (Ph*C*H_2_), 71.8 (Ph*C*H_2_), 70.9 (C-1'), 70.7, 70.1 (C-1'), 69.7, 68.2, 66.4 (C-6'), 64.2 (C-6'), 27.0 (*t-*Bu), 19.3 (CH_3_), 19.2 (CH_3_); MS (ESI): 2383.08 [M + Na]^+^; anal. calcd for C_148_H_158_O_23_Si_2_ (2361.06): C, 75.29; H, 6.75; found: C, 75.29; H, 6.79.

**Synthesis of olefin 15.** To a suspension of methyltriphenylphosphonium bromide (895 mg, 2.50 mmol) in benzene (20 mL) a 2.5 M solution of BuLi in hexane (0.95 mL, 2.30 mmol) was added and the mixture was stirred at rt for 90 min. A solution of aldehyde **7** (298 mg, 0.27 mmol) in benzene (5 mL) was added, the mixture was stirred for another 45 min, quenched with water (100 μL), concentrated, and the residue was purified by column chromatography (hexane–ethyl acetate, 15:1 → 10:1) to afford **15** (172 mg, 58%) as a foam. [α]_D_^20^ 24.0; ^1^H NMR δ 7.67*–*7.64 (m, 4H, Ar), 7.35*–*7.18 (m, 36H, Ar), 5.77*–*5.72 (m, 2H, H-1,6), 5.12*–*5.09 (m, 1H, H-7), 5.02*–*5.00 (m, 1H, H-7), 4.82*–*4.50 (m, 11H, PhC*H_2_*), 4.45*–*4.43 (m, 3H, H-3',5,PhC*H*), 4.30 (t, 1H, *J*_4,3_ = *J*_4,5_ = 7.4 Hz, H-4'), 4.05*–*4.02 (m, 1H, H-5'), 3.94 (dd, 1H, *J*_6,5_ = 4.8 Hz, *J*_6,6’_ = 11.1 Hz, H-6'), 3.90*–*3.87 (m, 2H, H-3,6'), 3.69 (d, 1H, *J*_gem_ = 11.0 Hz, H-1'), 3.50 (d, 1H, *J*_gem_ = 11.0 Hz, H-1'), 3.44 (dd, 1H, *J*_2,1_ = 3.7 Hz, *J*_2,3_ = 9.7 Hz, H-2), 3.16 (dd, 1H, *J*_4,3_ = 9.4 Hz, *J*_4,5_ = 9.5 Hz, H-4), 1.06 (s, 9H, *t-*Bu); ^13^C NMR δ 138.9, 138.6, 138.4, 138.3, 138.0, 135.7, 135.6, 135.6, 133.5, 133.3, 129.6, 129.6, 128.3–127.5 (Ar), 117.0 (C-7), 104.3 (C-2'), 89.2 (C-1), 83.8 (C-3'), 82.5 (C-4), 82.3 (C-4'), 81.6 (C-3), 81.2 (C-5'), 80.0 (C-2), 75.6 (Ph*C*H_2_), 74.8 (Ph*C*H_2_), 73.4 (Ph*C*H_2_), 72.9 (Ph*C*H_2_), 72.6 (Ph*C*H_2_), 72.3 (Ph*C*H_2_), 71.4 (C-1',5), 64.9 (C-6'), 26.9 (*t-*Bu-*C*H_3_), 19.3 (*t-*Bu-*C*); anal. calcd for C_71_H_76_O_10_Si (1117.47): C, 76.31; H, 6.86; found: C, 76.42; H, 6.99.

**Ozonolytic cleavage of the double bond in sucrose dimer 11. Determination of the configuration at the carbinol center***.* Ozone was passed through a cooled solution of **11** (51 mg, 0.023 mmol) in CH_2_Cl_2_ (10 mL) until the blue color persisted (10 min). Dimethyl disulfide (210 μL) was added, the mixture was stirred for 10 min, concentrated, and the residue was dissolved in methanol (10 mL). Sodium borohydride (40 mg) was added, the mixture was stirred for 1 h, concentrated, and the crude product was purified by column chromatography (hexane–ethyl acetate, 7:3) to afford sucrose **6** (16 mg, 63%) and diol **14** (18 mg, 69%), both as foam.

**Synthesis of diols 14 and 16.** To a solution of **15** (145 mg, 0.13 mmol) in THF (10 mL), *tert-*butyl alcohol (500 μL), water (50 μL), NMO (80 mg, 0.68 mmol), and OsO_4_ (8 wt % in *t-*BuOH, 150 μL, 0.035 mmol) were added, and the mixture was stirred at rt for 18 h. Saturated aq Na_2_S_2_O_3_ (0.2 mL) was added, the mixture was stirred for 1 h at rt, concentrated, and the products were isolated by column chromatography (hexane–ethyl acetate, 5:1 → 3:1) to afford of **14** (60 mg; 40%) and **16** (58 mg 39%), both as foam.

Data for **14**: [α]_D_^20^ 32.6; ^1^H NMR δ 7.64–7.62 (m, 4H, aryl-H), 7.36–7.14 (m, 36H, Ar), 6.04 (d, 1H, *J*_1,2_ = 3.8 Hz, H-1), 4.95–4.39 (m, 10H, PhC*H_2_*), 4.47–4.45 (m, 4H, H-3',4',PhC*H_2_*), 4.14 (dd, 1H, *J*_5,4_ = 10.2, *J_5,6_* = 4.1 Hz, H-5), 4.03 (dd, 1H, *J*_6,5_ = 3.4, *J*_6,6’_ = 11.6 Hz, H-6'), 3.93*–*3.89 (m, 2H, H-3,5'), 3.84*–*3.79 (m, 2H, H-6,6'), 3.65*–*3.62 (m, 2H, H-1',7), 3.55 (d, 1H, *J*_gem_ = 10.8 Hz, H-1'), 3.49*–*3.43 (m, 3H, H-2,4,7), 1.07 (s, 9H, *t-*Bu); ^13^C NMR δ 138.6, 138.2, 138.0, 137.9, 137.8, 137.3, 135.7, 135.4, 133.0, 132.6, 129.8, 129.7, 128.7–127.6 (Ar), 104.3 (C-2'), 88.2 (C-1), 82.9 (C-3'), 82.0 (C-3), 80.3 (C-5'), 80.1 (C-2,4'), 79.0 (C-4), 75.5 (Ph*C*H_2_), 74.7 (Ph*C*H_2_),73.4 (Ph*C*H_2_), 73.1 (Ph*C*H_2_), 72.8 (Ph*C*H_2_), 72.3 (C-1'), 72.2 (C-6), 71.9 (Ph*C*H_2_), 71.3 (C-5), 63.0 (C-6'), 62.9 (C-7), 26.9 (*t-*Bu-*C*H_3_), 19.2 (*t-*Bu-*C*); anal. calcd for C_71_H_78_O_12_Si (1151.49): C, 74.06; H, 6.83; found: C, 74.00; H, 6.79.

Data for **16**: [α]_D_^20^ 21.6; ^1^H NMR δ 7.64*–*7.62 (m, 4H, Ar), 7.37*–*7.16 (m, 36H, aryl-H), 6.04 (d, 1H, *J*_1,2_ = 3.6 Hz, H-1), 4.93*–*4.40 (m, 12H, PhC*H_2_*), 4.40 (d, 1H, *J* = 6.5 Hz, H-3'), 4.32 (t, 1H, *J*_4,3_ = *J*_4,5_ = 6.7 Hz, H-4'), 4.03*–*3.99 (m, 2H, H-5,6'), 3.95 (t, 1H, *J*_3,2_ = *J*_3,4_ = 9.4 Hz, H-3), 3.92*–*3.91 (m, 1H, H-5'), 3.81 (dd, 1H, *J*_6,5_ = 4.0 Hz, *J*_6,6’_ = 11.4 Hz, H-6'), 3.74*–*3.72 (m, 1H, H-6), 3.70*–*3.66 (m, 2H, H-1',4), 3.55 (d, 1H, *J*_gem_ = 10.9 Hz, H-1'), 3.48*–*3.42 (m, 3H, H-2,7,7), 1.07 (s, 9H, *t-*Bu); ^13^C NMR δ 138.8, 138.5, 138.3, 137.9, 137.7, 137.5, 135.7, 135.5, 132.9, 132.7, 129.8, 129.8, 128.4–127.5 (Ar), 104.8 (C-2'), 89.3 (C-1), 83.2 (C-3'), 81.4 (C-3), 81.1 (C-4'), 80.8 (C-5'), 79.9 (C-2), 77.3 (C-4), 75.5 (Ph*C*H_2_), 75.1 (Ph*C*H_2_), 73.5 (Ph*C*H_2_), 73.2 (Ph*C*H_2_), 72.5 (Ph*C*H_2_), 72.0 (Ph*C*H_2_), 71.9 (C-5), 71.6 (C-1'), 68.9 (C-6), 64.5 (C-7), 63.1 (C-6'), 26.9 (*t-*Bu-*C*H_3_), 19.2 (*t-*Bu-*C*); anal. calcd for C_71_H_78_O_12_Si (1151.49): C, 74.06; H, 6.83; found: C, 73.94; H, 6.66.

Both compounds were further characterized as diacetates: **14-Ac** and **16-Ac**.

Data for **14-Ac**: [α]_D_^20^ 33.3; ^1^H NMR δ 7.66*–*7.64 (m, 4H, Ar), 7.34*–*7.21 (m, 36H, aryl-H), 5.82 (d, 1H, *J*_1,2_ = 3.6 Hz, H-1), 5.48 (m, 1H, H-6), 4.93*–*4.55 (m, 10H, PhC*H_2_*), 4.46*–*4.42 (m, 3H, H-3', PhC*H_2_*), 4.15 (dd, 1H, *J*_5,4_ = 10.3 Hz, *J*_5,6_ = 1.4 Hz, H-5), 4.03*–*3.96 (m, 3H, H-5', H-6', H-7), 3.91 (dd, 1H, *J*_6,5_ = 4.7, *J*_6,6’_ = 10.9 Hz, H-6'), 3.88 (t, 1H, *J*_3,2_ = *J*_3,4_ = 9.2 Hz, H-3), 3.67 (d, 1H, *J*_1,1_ = 11.0 Hz, H-1'), 3.51 (d, 1H, *J*_1,1_ = 11.0 Hz, H-1'), 3.49 (dd, 1H, *J*_4,3_ = 9.2 Hz, *J*_4,5_ = 10.3 Hz, H-4), 3.39 (dd, 1H, *J*_2,1_ = 3.6 Hz, *J*_2,3_ = 9.7 Hz, H-2), 1.96 (s, 3H, CH_3_), 1.84 (s, 3H, CH_3_), 1.05 (s, 9H, *t-*Bu); ^13^C NMR δ 170.5 (C=O), 169.8 (C=O), 138.6, 138.4, 138.2, 138.1, 138.0, 137.9, 135.7, 135.5, 133.3, 133.0, 129.7, 129.6, 128.3-127.5 (Ar), 104.2 (C-2'), 89.0 (C-1), 83.7 (C-3'), 82.0 (C-3), 81.9 (C-4'), 81.0 (C-5'), 79.8 (C-2), 77.6 (C-4), 75.6 (Ph*C*H_2_), 74.6 (Ph*C*H_2_), 73.3 (Ph*C*H_2_), 73.1 (Ph*C*H_2_), 72.8 (Ph*C*H_2_), 72.1 (Ph*C*H_2_), 71.4 (C-1'), 71.1 (C-6), 70.9 (C-5), 64.6 (C-6'), 63.2 (C-7), 26.9, 20.9, 20.8, 19.2; anal. calcd for C_75_H_82_O_14_Si × ½H_2_O (1253.57): C, 72.38; H, 6.72; found: C, 72.51; H, 6.36. HRMS (ESI) calc. for C_75_H_86_NO_14_Si [M + NH_4_]^+^: 1252.5818; found: 1252.5825.

Data for **16-Ac**: [α]_D_^20^ 13.1; ^1^H NMR (CDCl_3_) δ 7.65*–*7.63 (m, 4H, Ar), 7.34*–*7.20 (m, 36H, Ar), 6.09 (d, 1H, *J*_1,2_ = 3.5 Hz, H-1), 5.48 (ddd, 1H, *J* = 1.5 Hz, 2.9 Hz, 9.2 Hz, H-6), 4.88*–*4.42 (m, 12H, PhC*H_2_*), 4.42*–*4.38 (m, 2H, H-3', H-4'), 4.28 (dd, 1H, *J*_7,6_ = 9.1 Hz, *J*_7,7_ = 12.0 Hz, H-7), 4.11 (dd, 1H, *J*_5,4_ = 10.1 Hz, *J*_5,6_ = 1.3 Hz, H-5), 4.06 (dd, 1H, *J*_7,6_ = 3.1 Hz, *J*_7,7_ = 12.0 Hz, H-7), 4.02 (dd, 1H, *J*_6,5_ = 4.2 Hz, *J*_6,6’_ = 11.4 Hz, H-6'), 3.97*–*3.93 (m, 2H, H-3, H-5'), 3.85 (dd, 1H, *J*_6,5_ = 4.1 Hz, *J*_6,6’_ = 11.4 Hz, H-6'), 3.74 (d, 1H, *J*_1,1_ = 10.8 Hz, H-1'), 3.53 (d, 1H, *J*_1,1_ = 10.8 Hz, H-1'), 3.48 (dd, 1H, *J*_2,1_ = 3.5 Hz, *J*_2,3_ = 9.7 Hz, H-2), 3.33 (dd, 1H, *J*_4,3_ = 8.9 Hz, *J*_4,5_ = 10.1 Hz, H-4), 2.08 (s, 3H, CH_3_), 1.95 (s, 3H, CH_3_), 1.06 (s, 9H, *t-*Bu); ^13^C NMR (CDCl_3_) δ 170.3 (C=O), 170.2 (C=O), 138.5, 138.3, 138.3, 138.2, 137.8, 137.7, 135.6, 135.5, 133.2, 132.7, 129.7, 129.6, 128.5–127.5 (Ar), 104.6 (C-2'), 89.1 (C-1), 83.7 (C-3'), 81.6 (C-3), 81.3 (C-4'), 80.8 (C-5'), 79.8 (C-2), 76.9 (C-4), 75.6 (Ph*C*H_2_), 75.0 (Ph*C*H_2_), 73.5 (Ph*C*H_2_), 73.3 (Ph*C*H_2_), 72.8 (Ph*C*H_2_), 71.8 (Ph*C*H_2_), 71.4 (C-1'), 70.2 (C-5), 69.0 (C-6), 64.8 (C-7), 63.7 (C-6'), 26.9, 21.0, 20.8, 19.2; anal. calcd for C_75_H_82_O_14_Si × ½H_2_O (1253.57): C, 72.38; H, 6.72; found: C, 72.34; H, 6.29. HRMS (ESI) calc. for C_75_H_86_NO_14_Si [M + NH_4_]^+^: 1252.5818; found: 1252.5819.

## Supporting Information

File 1 The ^1^H and ^13^C NMR spectra of all new compounds (**9**–**16Ac**).
