# Supplementary material for: Synthesis of a sucrose dimer with enone tether; a study on its functionalization
Source: Beilstein J Org Chem. 2014 May 28;10:1246–54. doi: 10.3762/bjoc.10.124 (PMC4077423; doi:10.3762/bjoc.10.124)
Supplement: File 1 — The 1H and 13C NMR spectra of all new compounds (9–16Ac). [file Beilstein_J_Org_Chem-10-1246-s001.pdf]

Supporting Information

for

Synthesis of a sucrose dimer with enone tether; a study on its functionalization

Zbigniew Pakulski, Norbert Gajda, Magdalena Jawiczuk, Jadwiga Frelek, Piotr Cmoch and Sławomir Jarosz*

Address: Institute of Organic Chemistry, Polish Academy of Sciences, ul. Kasprzaka 44/52, 01-224 Warsaw, Poland

Email: Sławomir Jarosz - slawomir.Jarosz@icho.edu.pl

* Corresponding author

The ^{1}H and ^{13}C NMR spectra of all new compounds (9–16Ac).

Table of contents:

	page
^{1}H NMR spectrum of **9**	....S2
^{13}C NMR spectrum of **9**	....S3
^{1}H NMR spectrum of **10**	....S4
^{13}C NMR spectrum of **10**	....S5
^{1}H NMR spectrum of **11**	....S6
^{13}C NMR spectrum of **11**	....S7
^{1}H NMR spectrum of **12**	....S8
^{13}C NMR spectrum of **12**	....S9
^{1}H NMR spectrum of **13**	......................................S10
^{13}C NMR spectrum of **13**	......................................S11
^{1}H NMR spectrum of **14**	......................................S12
^{13}C NMR spectrum of **14**	......................................S13
^{1}H NMR spectrum of **14-Ac**	......................................S14
^{13}C NMR spectrum of **14-Ac**	......................................S15
^{1}H NMR spectrum of **15**	......................................S16
^{13}C NMR spectrum of **15**	......................................S17
^{1}H NMR spectrum of **16**	......................................S18
^{13}C NMR spectrum of **16**	......................................S19
^{1}H NMR spectrum of **16-Ac**	......................................S20
^{13}C NMR spectrum of **16-Ac**	......................................S21

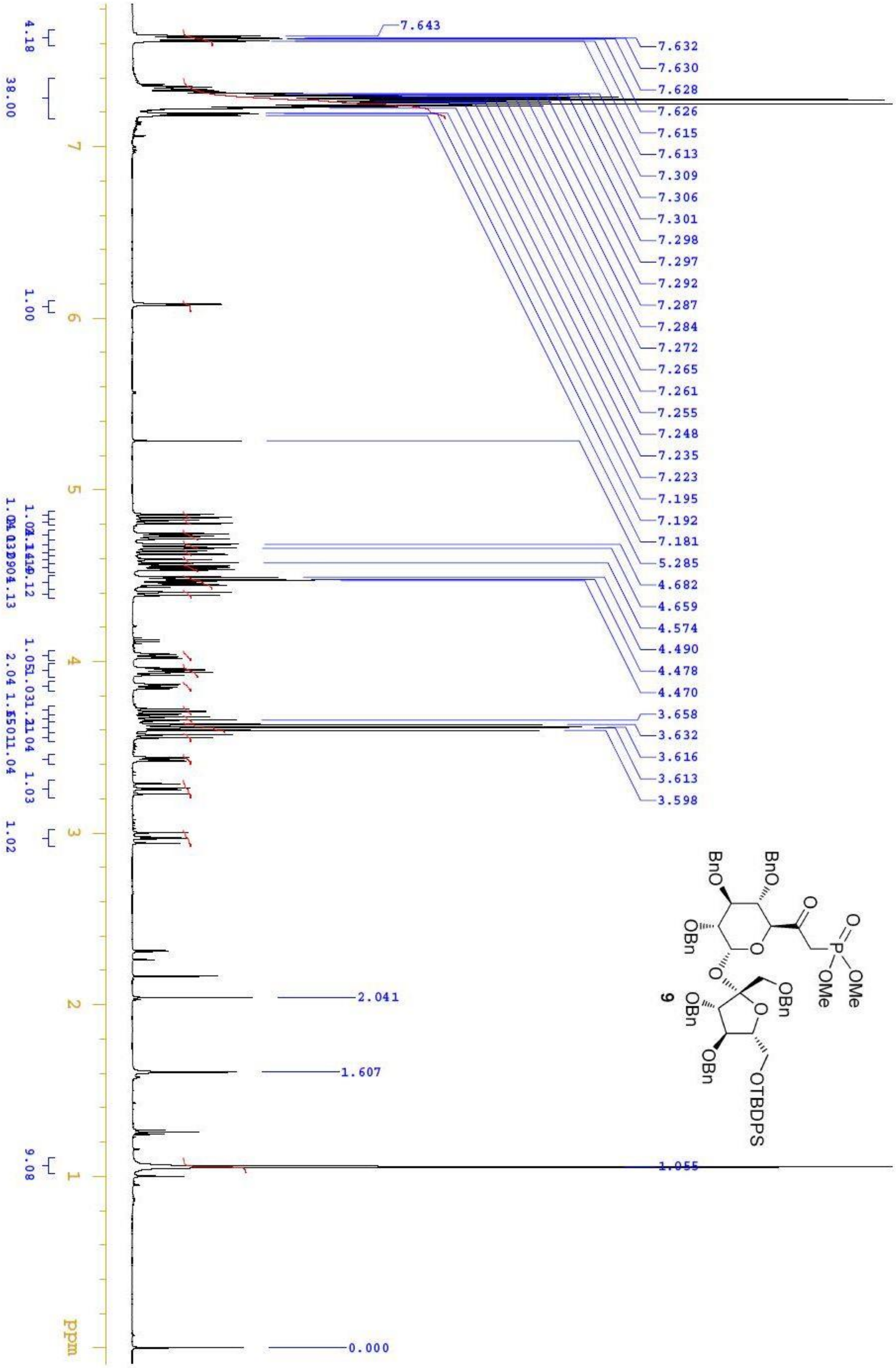
7.643
7.632
7.630
7.628
7.626
7.615
7.613
7.309
7.306
7.301
7.298
7.297
7.292
7.287
7.284
7.272
7.265
7.261
7.255
7.248
7.235
7.223
7.195
7.192
7.181
5.285
4.682
4.659
4.574
4.490
4.478
4.470
3.658
3.632
3.616
3.613
3.598
2.041
1.607
1.055
0.000
4.18
38.00
1.00
9.08
1.02
1.03
ppm
BnO
BnO
OBn
O
OMe
OMe
P
OBn
OBn
OBn
OTBDPS
9

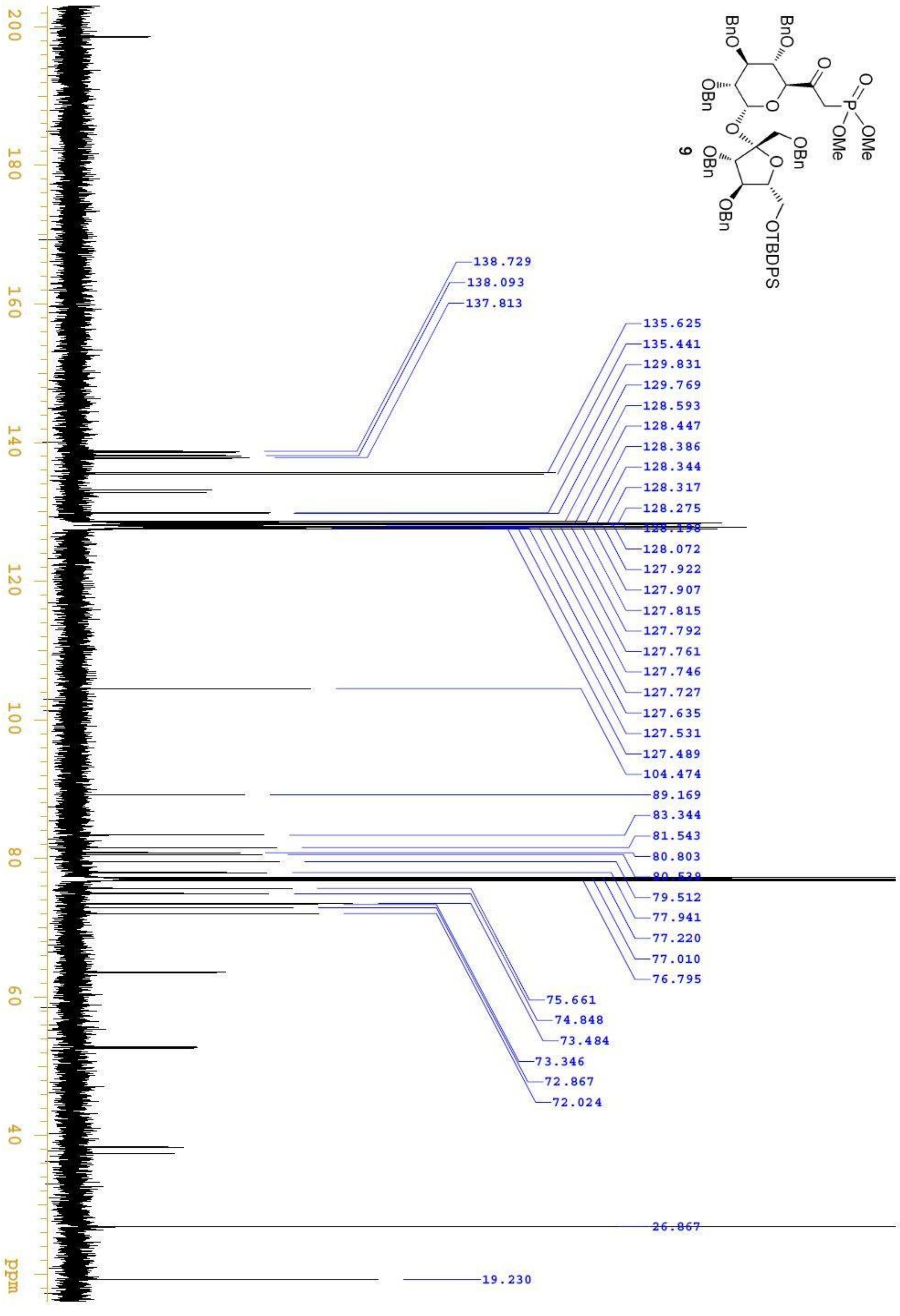

BnO
BnO
OBn
O
O
P
OMe
OMe
OBn
OBn
OBn
OTBDPS
9
138.729
138.093
137.813
135.625
135.441
129.831
129.769
128.593
128.447
128.386
128.344
128.317
128.275
128.198
128.072
127.922
127.907
127.815
127.792
127.761
127.746
127.727
127.635
127.531
127.489
104.474
89.169
83.344
81.543
80.803
79.512
77.941
77.220
77.010
76.795
75.661
74.848
73.484
73.346
72.867
72.024
26.867
19.230
200
180
160
140
120
100
80
60
40
ppm

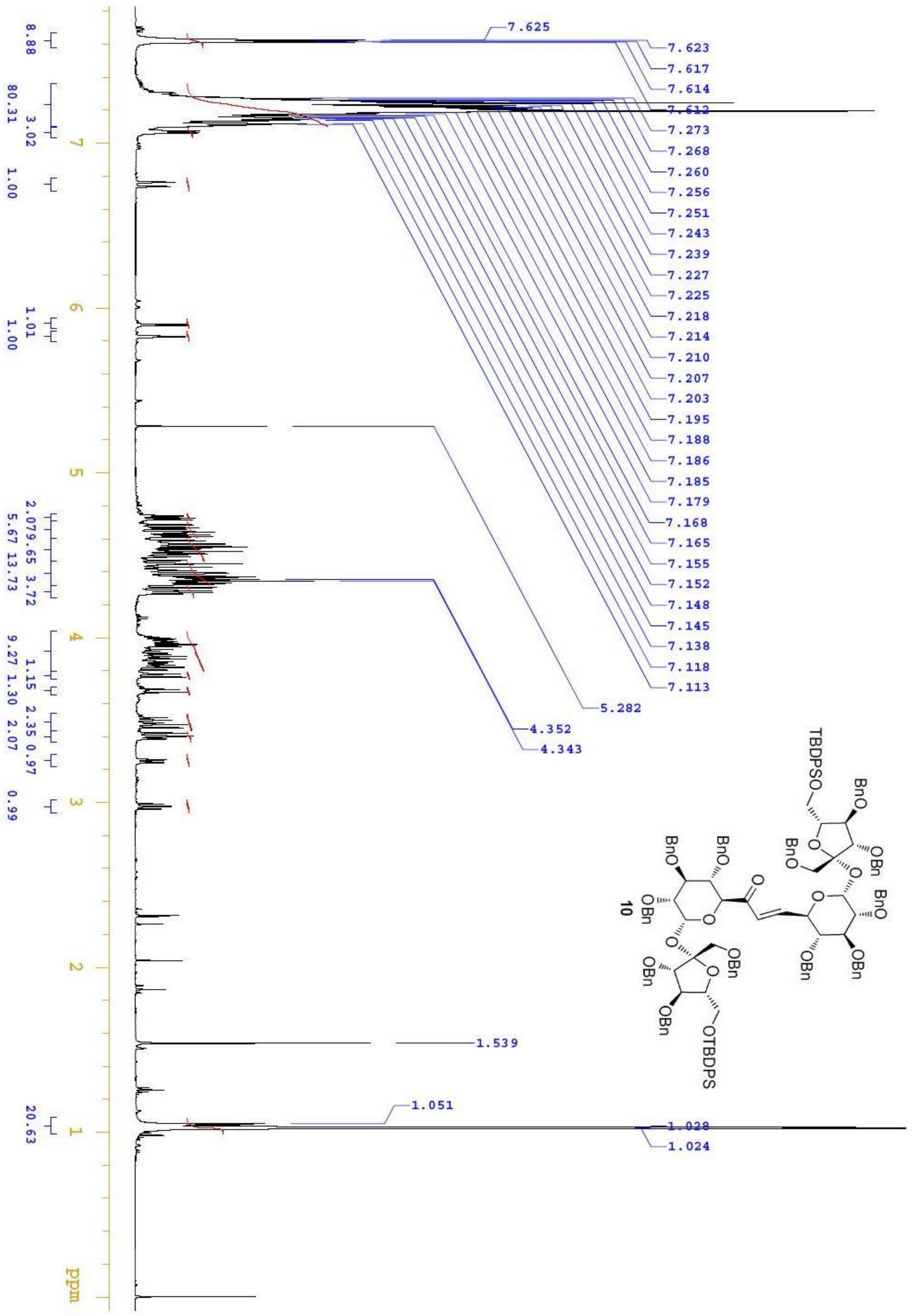
7.625
7.623
7.617
7.614
7.612
7.273
7.268
7.260
7.256
7.251
7.243
7.239
7.227
7.225
7.218
7.214
7.210
7.207
7.203
7.195
7.188
7.186
7.185
7.179
7.168
7.165
7.155
7.152
7.148
7.145
7.138
7.118
7.113
5.282
4.352
4.343
1.539
1.051
1.028
1.024
8.88
80.31
3.02
1.00
1.01
1.00
2.07
9.65
3.72
5.67
13.73
9.27
1.15
1.30
2.35
0.97
2.07
0.99
20.63
ppm
TBDPSO
BnO
OBn
OTBDPS
10

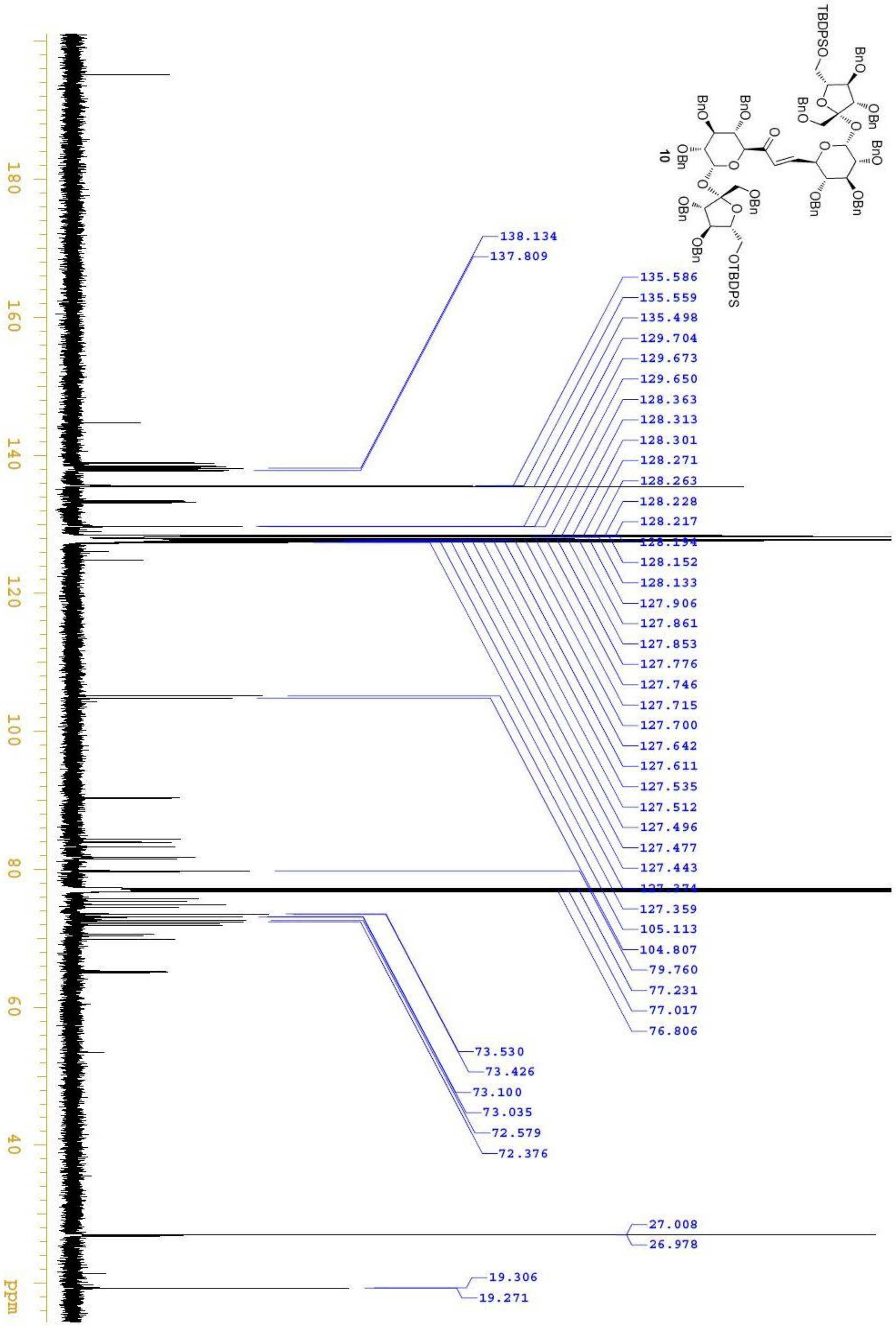

TBDPSO
BnO
OBn
BnO
OBn
OBn
OBn
O
BnO
BnO
OBn
10
OBn
OBn
OBn
OTBDPS
138.134
137.809
135.586
135.559
135.498
129.704
129.673
129.650
128.363
128.313
128.301
128.271
128.263
128.228
128.217
128.194
128.152
128.133
127.906
127.861
127.853
127.776
127.746
127.715
127.700
127.642
127.611
127.535
127.512
127.496
127.477
127.443
127.374
127.359
105.113
104.807
79.760
77.231
77.017
76.806
73.530
73.426
73.100
73.035
72.579
72.376
27.008
26.978
19.306
19.271
180
160
140
120
100
80
60
40
ppm

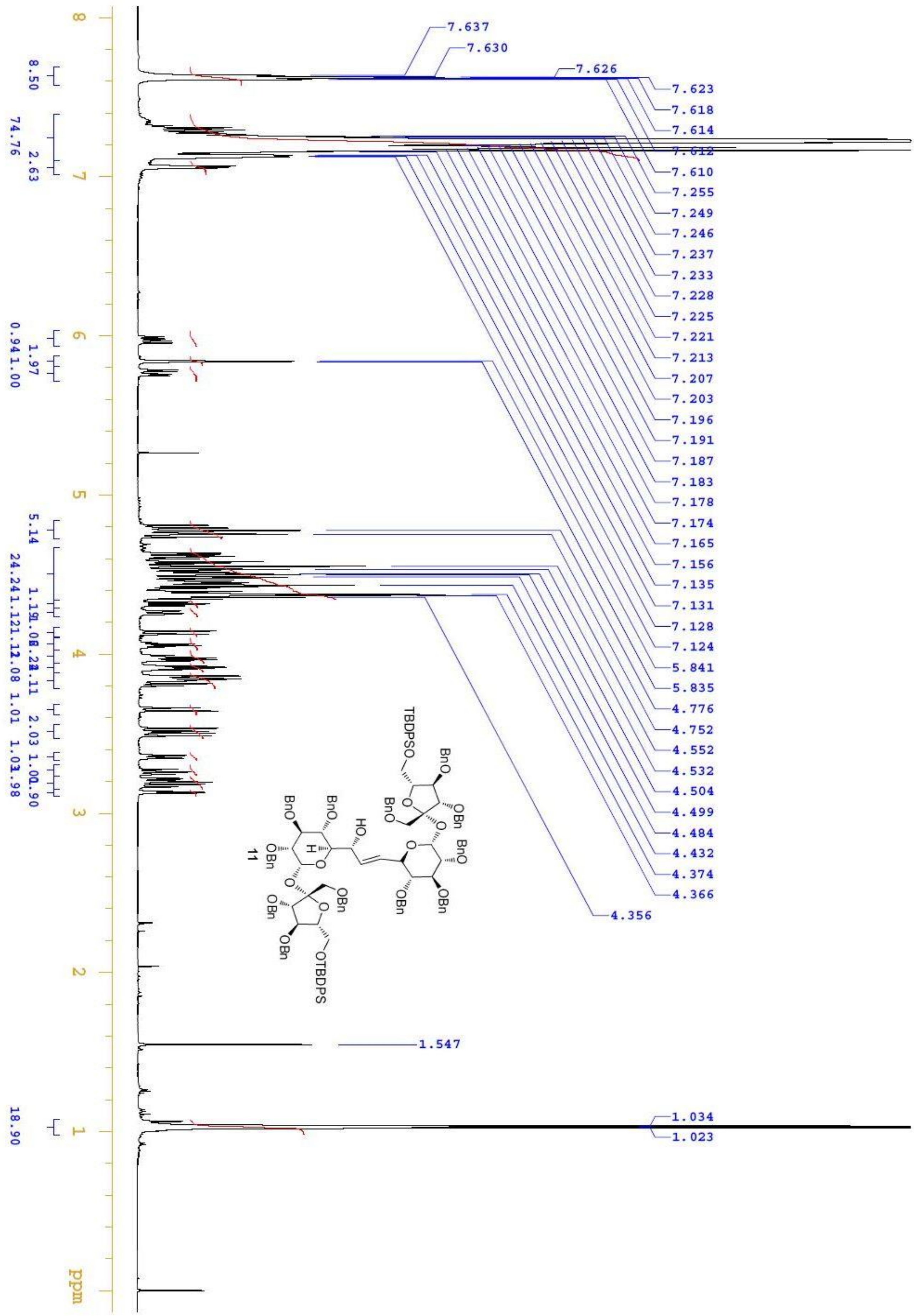
7.637
7.630
7.626
7.623
7.618
7.614
7.612
7.610
7.255
7.249
7.246
7.237
7.233
7.228
7.225
7.221
7.213
7.207
7.203
7.196
7.191
7.187
7.183
7.178
7.174
7.165
7.156
7.135
7.131
7.128
7.124
5.841
5.835
4.776
4.752
4.552
4.532
4.504
4.499
4.484
4.432
4.374
4.366
4.356
1.547
1.034
1.023
ppm
11
TBDPSO
OTBDPS
HO

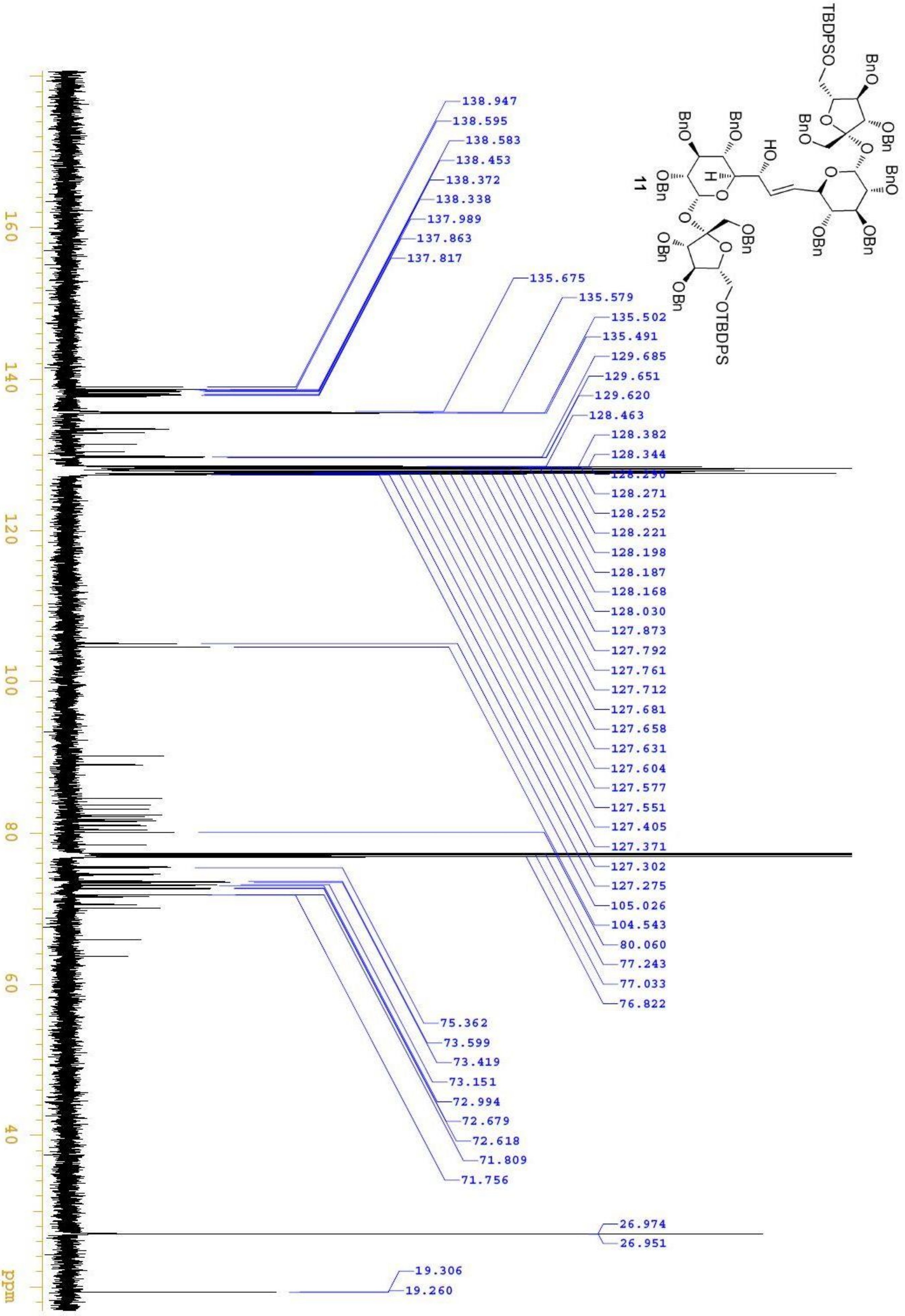
TBDPSO
BnO
BnO
OBn
BnO
BnO
BnO
HO
H
OBn
OBn
OBn
OBn
OBn
OTBDPS
11
138.947
138.595
138.583
138.453
138.372
138.338
137.989
137.863
137.817
135.675
135.579
135.502
135.491
129.685
129.651
129.620
128.463
128.382
128.344
128.290
128.271
128.252
128.221
128.198
128.187
128.168
128.030
127.873
127.792
127.761
127.712
127.681
127.658
127.631
127.604
127.577
127.551
127.405
127.371
127.302
127.275
105.026
104.543
80.060
77.243
77.033
76.822
75.362
73.599
73.419
73.151
72.994
72.679
72.618
71.809
71.756
26.974
26.951
19.306
19.260
160
140
120
100
80
60
40
ppm

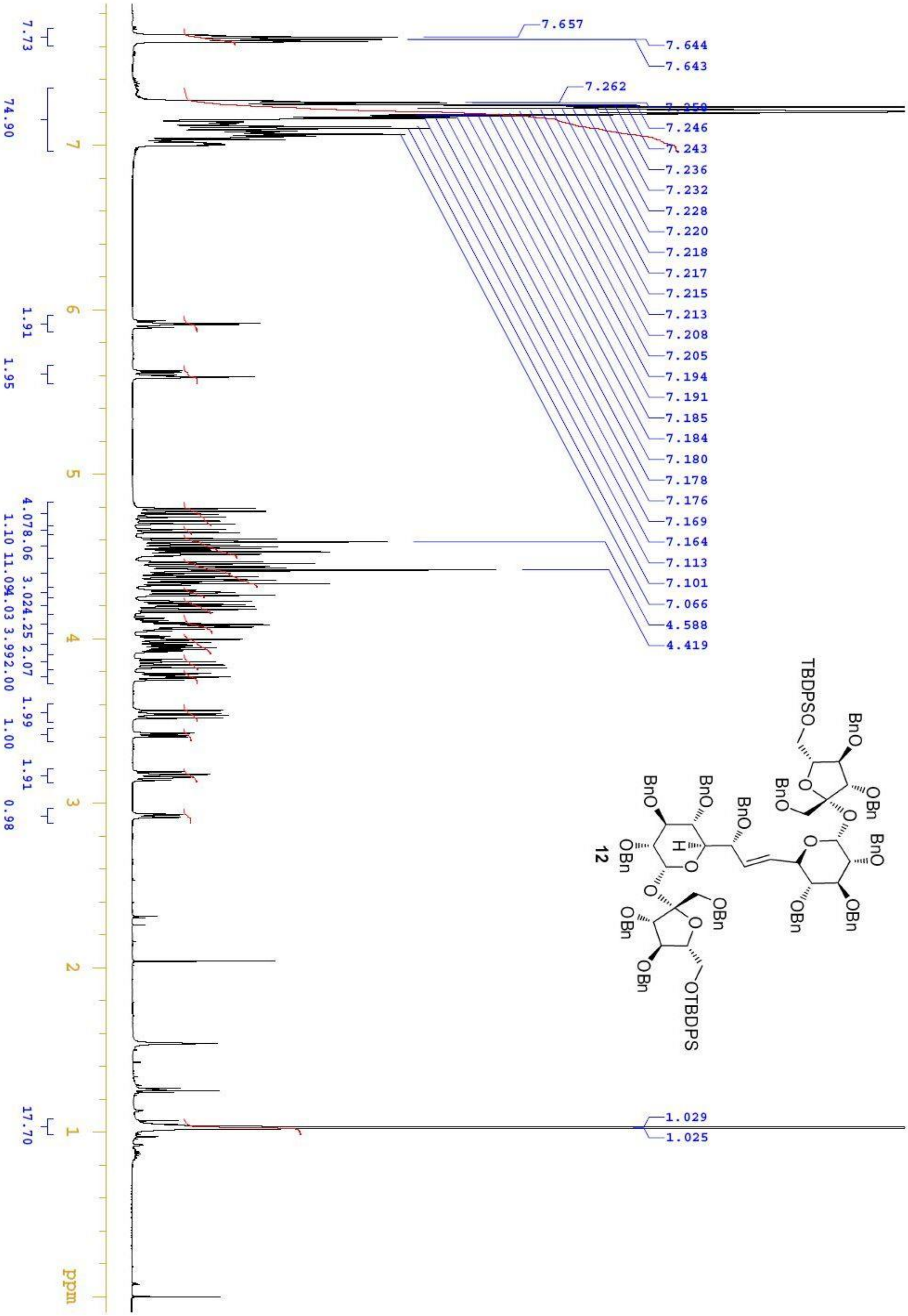
7.657
7.644
7.643
7.262
7.246
7.243
7.236
7.232
7.228
7.220
7.218
7.217
7.215
7.213
7.208
7.205
7.194
7.191
7.185
7.184
7.180
7.178
7.176
7.169
7.164
7.113
7.101
7.066
4.588
4.419
1.029
1.025
7.73
74.90
1.91
1.95
1.99
1.00
1.91
0.98
17.70
ppm
TBDPSO
BnO
OBn
OTBDPS
12

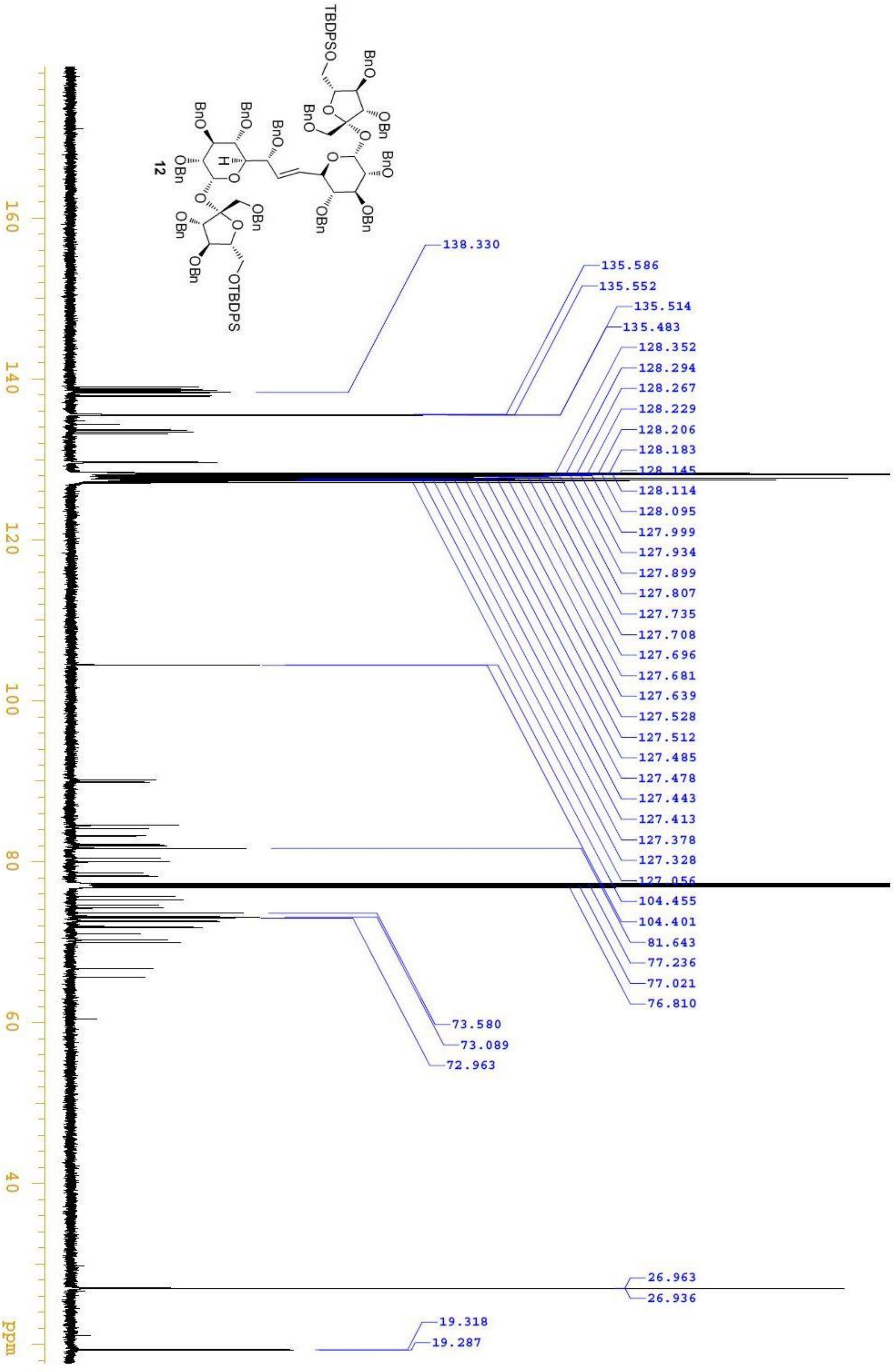
12
138.330
135.586
135.552
135.514
135.483
128.352
128.294
128.267
128.229
128.206
128.183
128.145
128.114
128.095
127.999
127.934
127.899
127.807
127.735
127.708
127.696
127.681
127.639
127.528
127.512
127.485
127.478
127.443
127.413
127.378
127.328
127.056
104.455
104.401
81.643
77.236
77.021
76.810
73.580
73.089
72.963
26.963
26.936
19.318
19.287
160
140
120
100
80
60
40
ppm

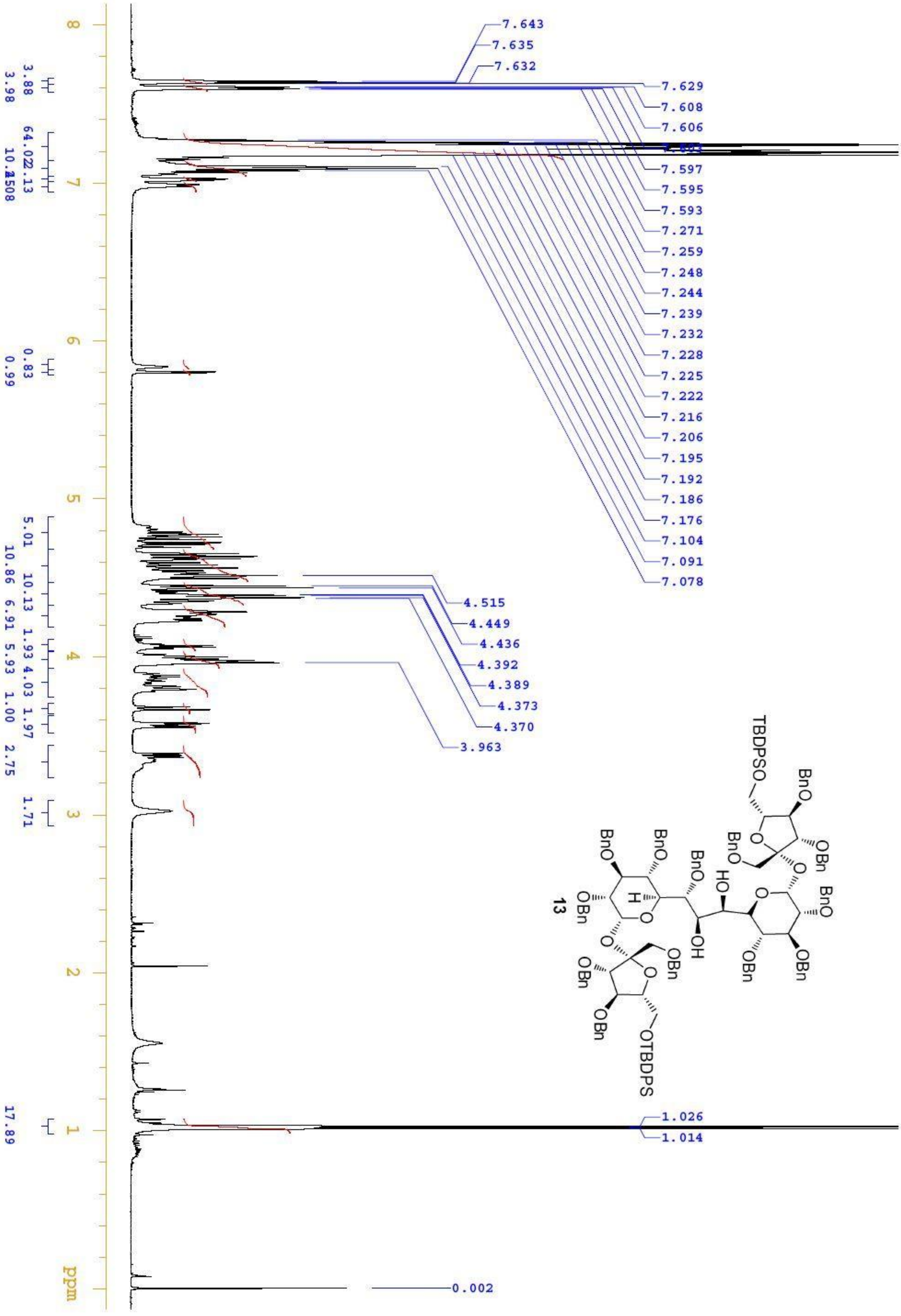

ppm
8
7
6
5
4
3
2
1
3.88
3.98
64.02
2.13
0.83
0.99
5.01
10.86
10.13
6.91
1.93
5.93
4.03
1.00
1.97
2.75
1.71
17.89
7.643
7.635
7.632
7.629
7.608
7.606
7.597
7.595
7.593
7.271
7.259
7.248
7.244
7.239
7.232
7.228
7.225
7.222
7.216
7.206
7.195
7.192
7.186
7.176
7.104
7.091
7.078
4.515
4.449
4.436
4.392
4.389
4.373
4.370
3.963
1.026
1.014
0.002
TBDPSO
BnO
OBn
HO
OH
OTBDPS
H
13

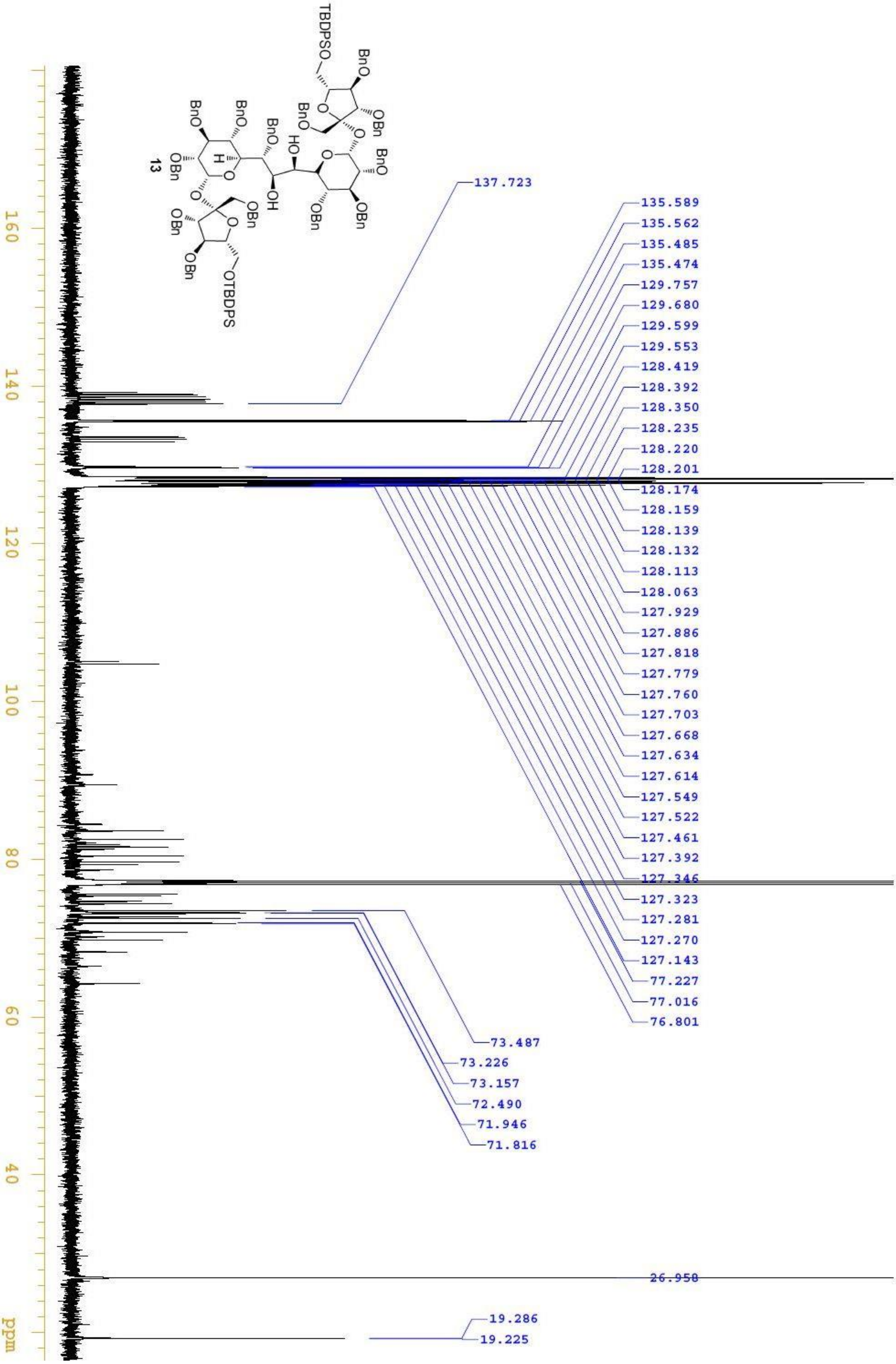
13
137.723
135.589
135.562
135.485
135.474
129.757
129.680
129.599
129.553
128.419
128.392
128.350
128.235
128.220
128.201
128.174
128.159
128.139
128.132
128.113
128.063
127.929
127.886
127.818
127.779
127.760
127.703
127.668
127.634
127.614
127.549
127.522
127.461
127.392
127.346
127.323
127.281
127.270
127.143
77.227
77.016
76.801
73.487
73.226
73.157
72.490
71.946
71.816
26.958
19.286
19.225
160
140
120
100
80
60
40
ppm

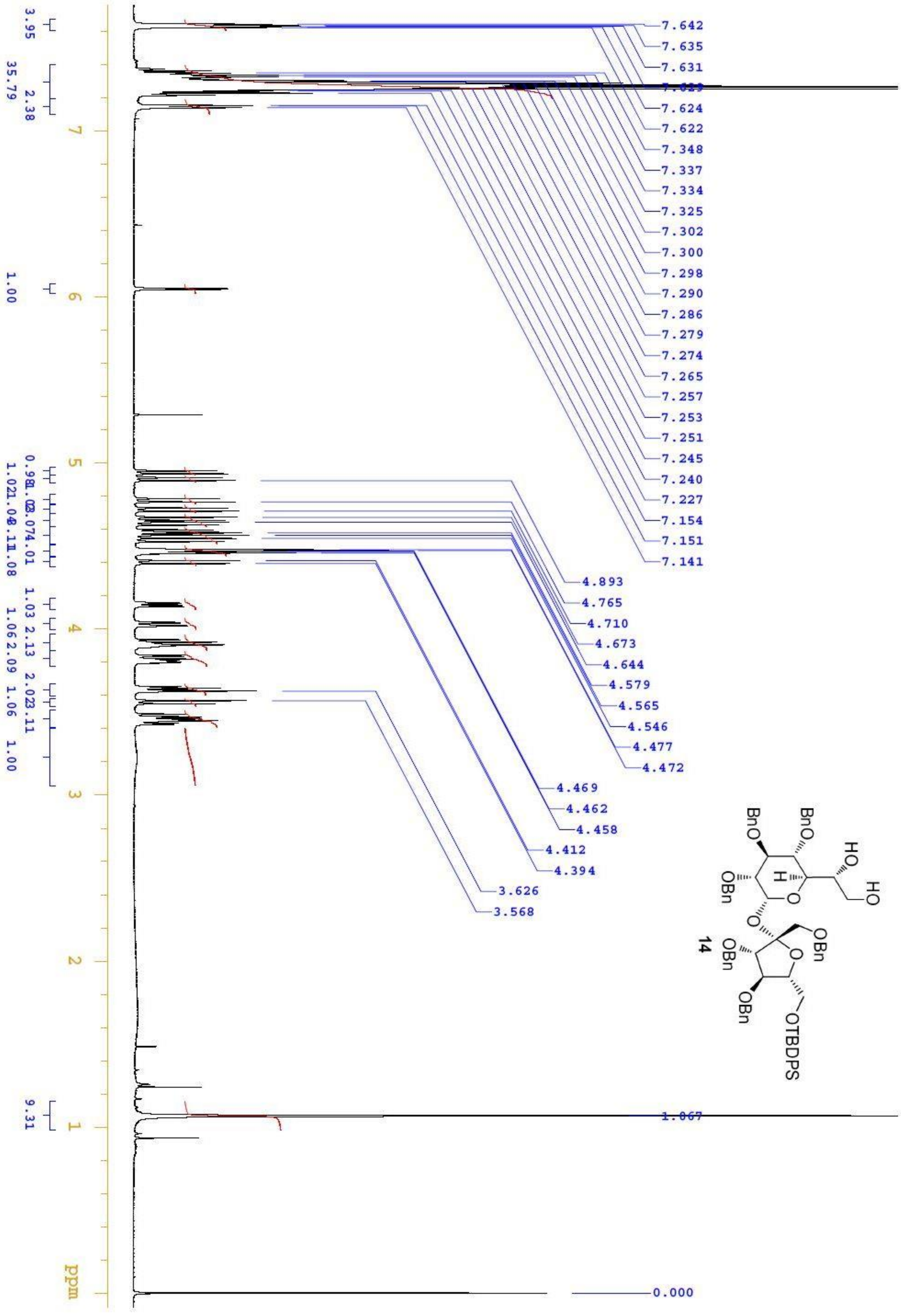
7.642
7.635
7.631
7.624
7.622
7.348
7.337
7.334
7.325
7.302
7.300
7.298
7.290
7.286
7.279
7.274
7.265
7.257
7.253
7.251
7.245
7.240
7.227
7.154
7.151
7.141
4.893
4.765
4.710
4.673
4.644
4.579
4.565
4.546
4.477
4.472
4.469
4.462
4.458
4.412
4.394
3.626
3.568
1.067
0.000
3.95
35.79
2.38
1.00
1.03
2.13
1.06
2.09
1.06
1.00
9.31
7
6
5
4
3
2
1
ppm
BnO
BnO
HO
HO
OBn
H
O
O
OBn
OBn
OBn
OTBDPS
14

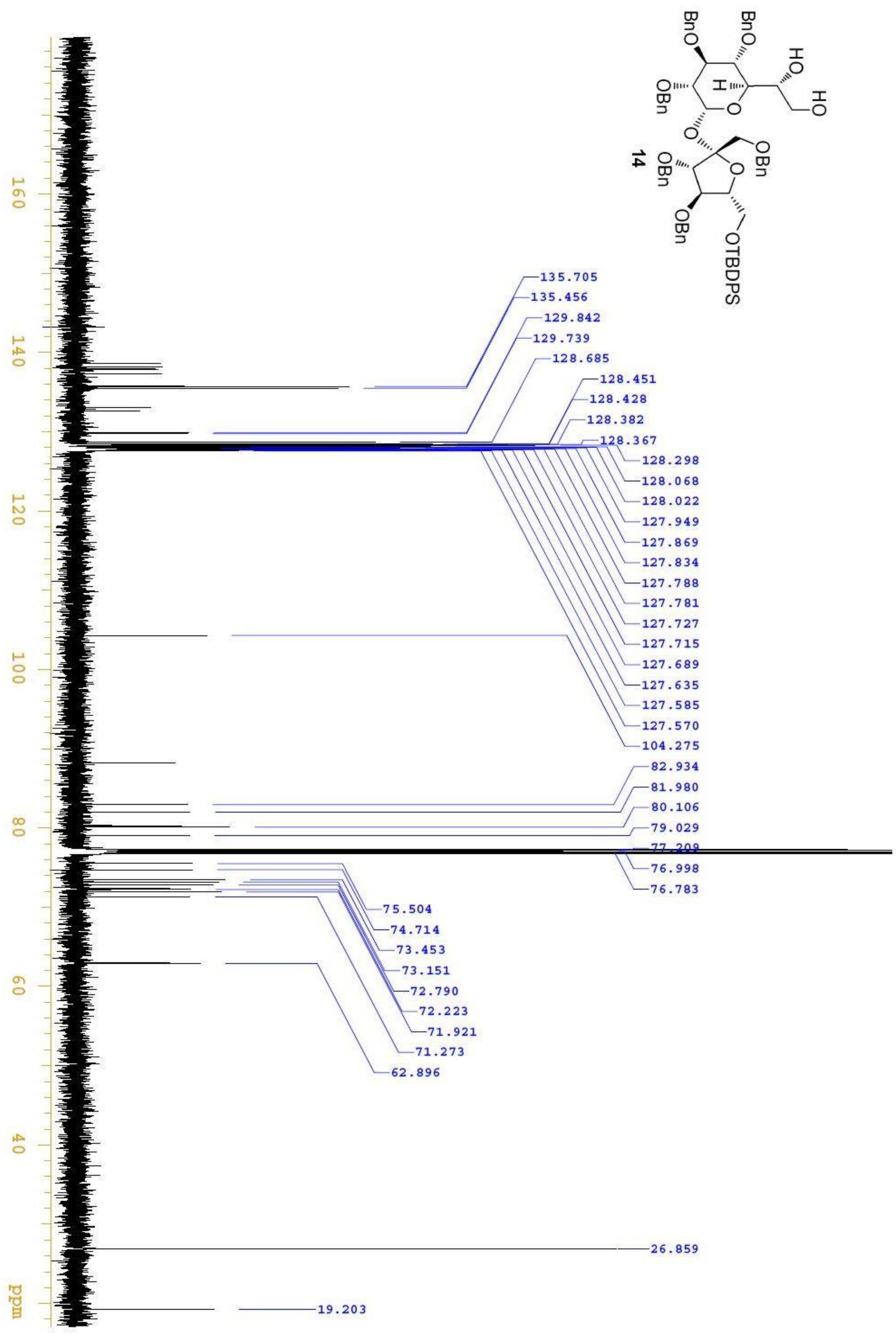
14
OTBDPS
135.705
135.456
129.842
129.739
128.685
128.451
128.428
128.382
128.367
128.298
128.068
128.022
127.949
127.869
127.834
127.788
127.781
127.727
127.715
127.689
127.635
127.585
127.570
104.275
82.934
81.980
80.106
79.029
77.209
76.998
76.783
75.504
74.714
73.453
73.151
72.790
72.223
71.921
71.273
62.896
26.859
19.203
ppm

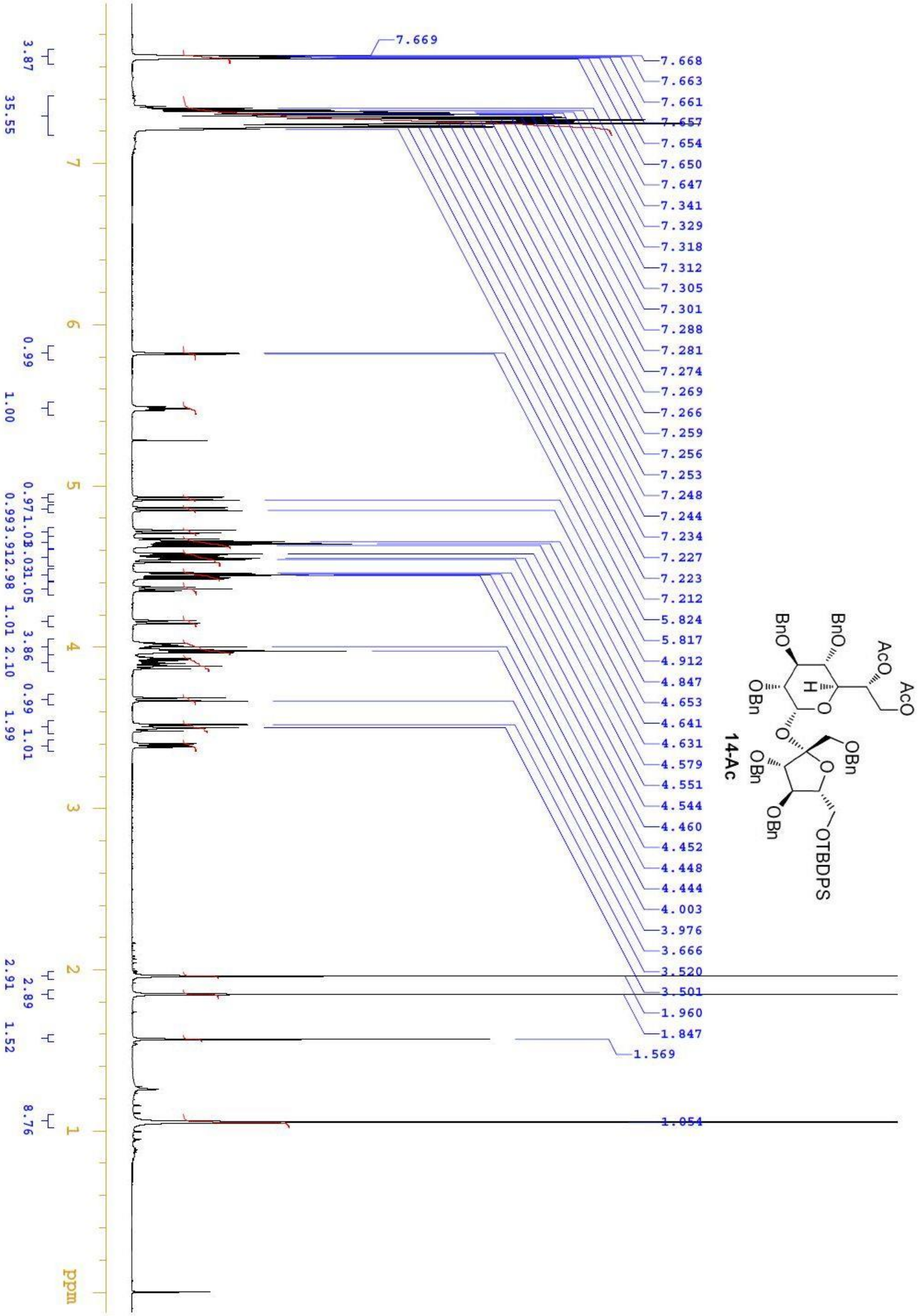
7.669
7.668
7.663
7.661
7.657
7.654
7.650
7.647
7.341
7.329
7.318
7.312
7.305
7.301
7.288
7.281
7.274
7.269
7.266
7.259
7.256
7.253
7.248
7.244
7.234
7.227
7.223
7.212
5.824
5.817
4.912
4.847
4.653
4.641
4.631
4.579
4.551
4.544
4.460
4.452
4.448
4.444
4.003
3.976
3.666
3.520
3.501
1.960
1.847
1.569
1.054
3.87
35.55
0.99
1.00
0.97
1.01
1.03
1.05
0.99
3.91
2.98
1.01
2.10
3.86
0.99
1.99
1.01
2.91
2.89
1.52
8.76
7
6
5
4
3
2
1
ppm
AcO
AcO
BnO
BnO
OBn
OBn
OBn
OBn
H
O
O
O
OTBDPS
14-Ac

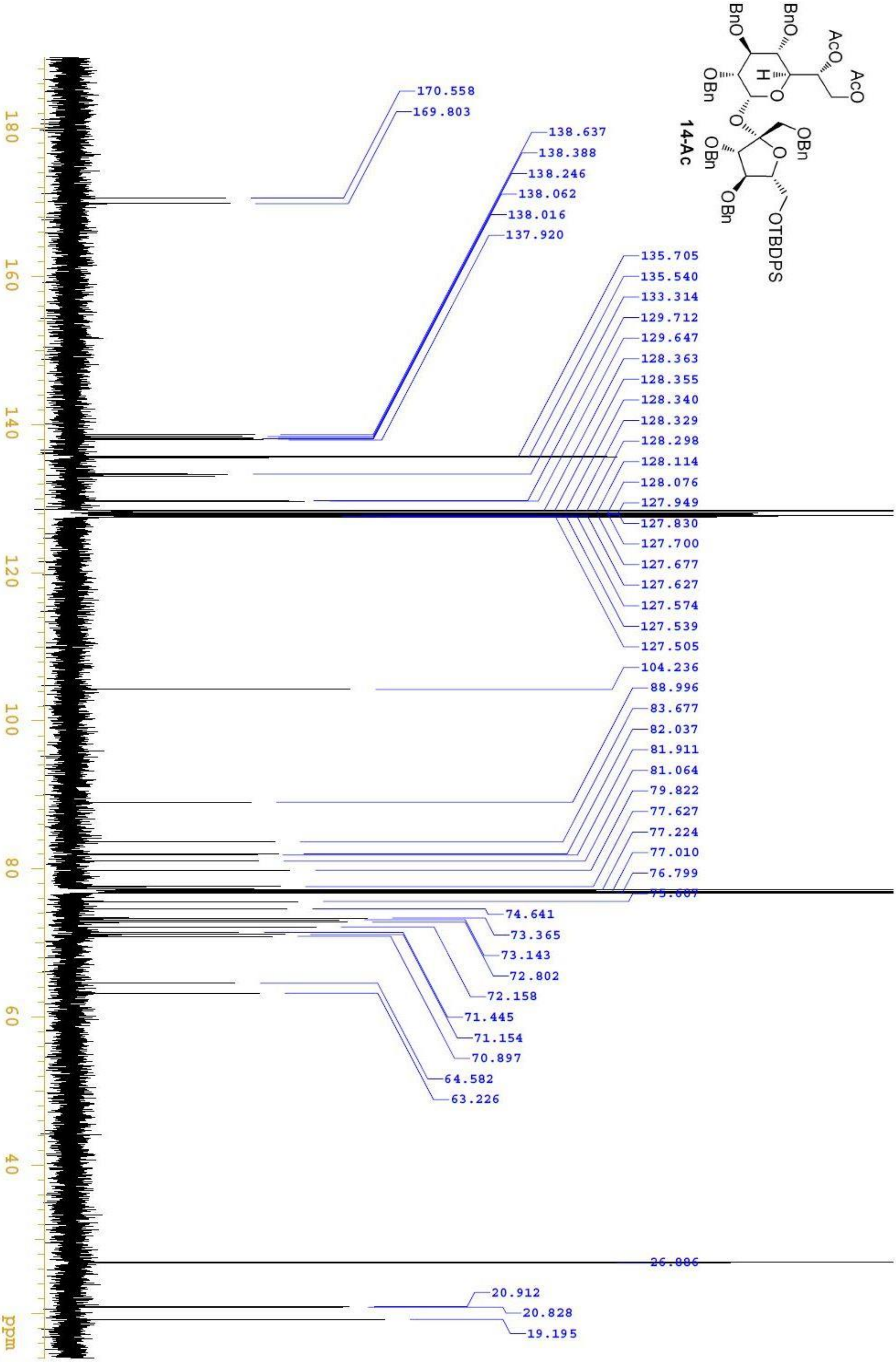

BnO
BnO
AcO
AcO
OBn
H
O
OBn
OBn
OBn
O
OTBDPS
14-Ac
170.558
169.803
138.637
138.388
138.246
138.062
138.016
137.920
135.705
135.540
133.314
129.712
129.647
128.363
128.355
128.340
128.329
128.298
128.114
128.076
127.949
127.830
127.700
127.677
127.627
127.574
127.539
127.505
104.236
88.996
83.677
82.037
81.911
81.064
79.822
77.627
77.224
77.010
76.799
74.641
73.365
73.143
72.802
72.158
71.445
71.154
70.897
64.582
63.226
26.886
20.912
20.828
19.195
180
160
140
120
100
80
60
40
ppm

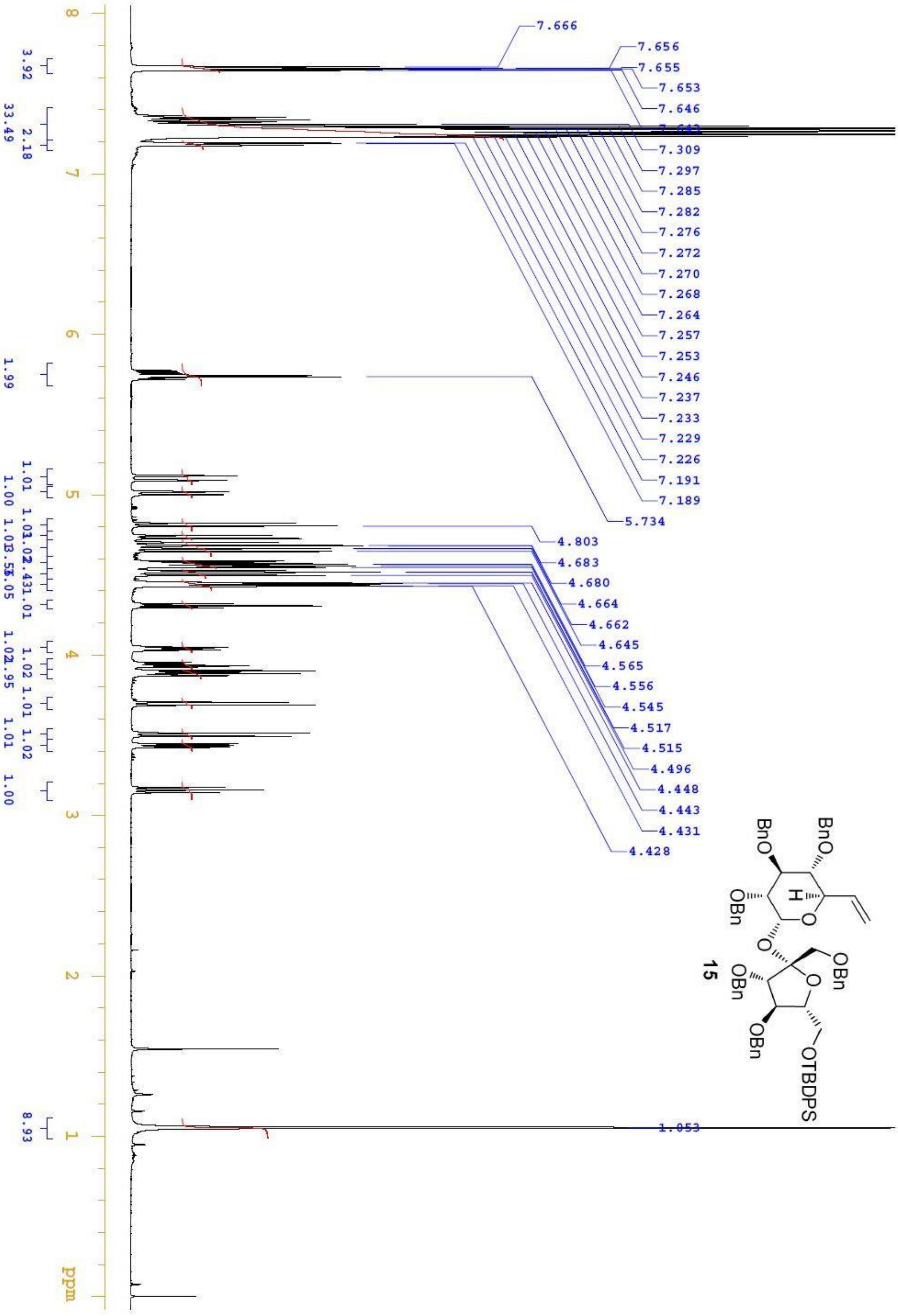
7.666
7.656
7.655
7.653
7.646
7.309
7.297
7.285
7.282
7.276
7.272
7.270
7.268
7.264
7.257
7.253
7.246
7.237
7.233
7.229
7.226
7.191
7.189
5.734
4.803
4.683
4.680
4.664
4.662
4.645
4.565
4.556
4.545
4.517
4.515
4.496
4.448
4.443
4.431
4.428
1.053
ppm
BnO
OBn
OTBDPS
15

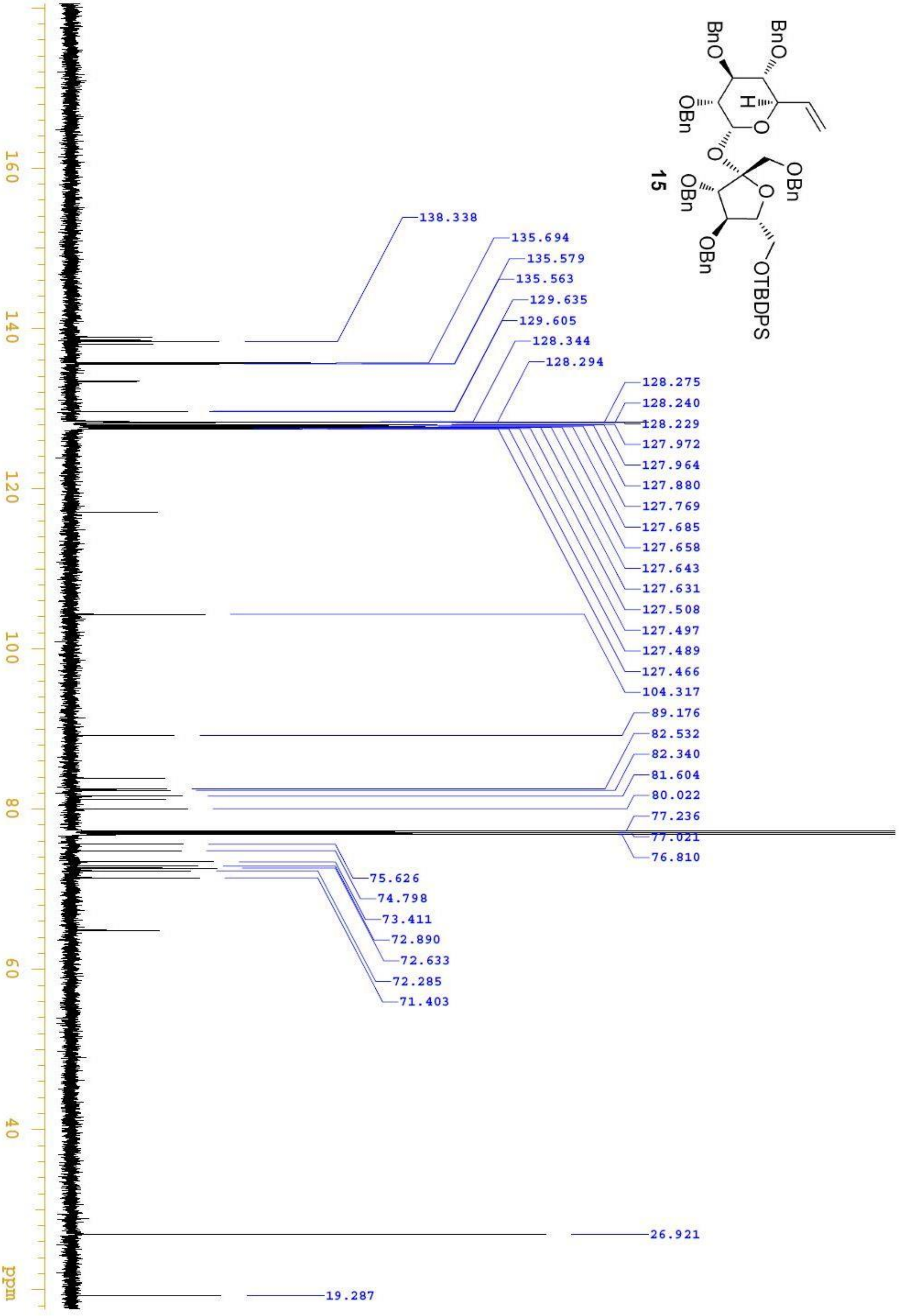

138.338
135.694
135.579
135.563
129.635
129.605
128.344
128.294
128.275
128.240
128.229
127.972
127.964
127.880
127.769
127.685
127.658
127.643
127.631
127.508
127.497
127.489
127.466
104.317
89.176
82.532
82.340
81.604
80.022
77.236
77.021
76.810
75.626
74.798
73.411
72.890
72.633
72.285
71.403
26.921
19.287
160
140
120
100
80
60
40
ppm
BnO
BnO
OBn
H
O
OBn
OBn
OBn
OTBDPS
15

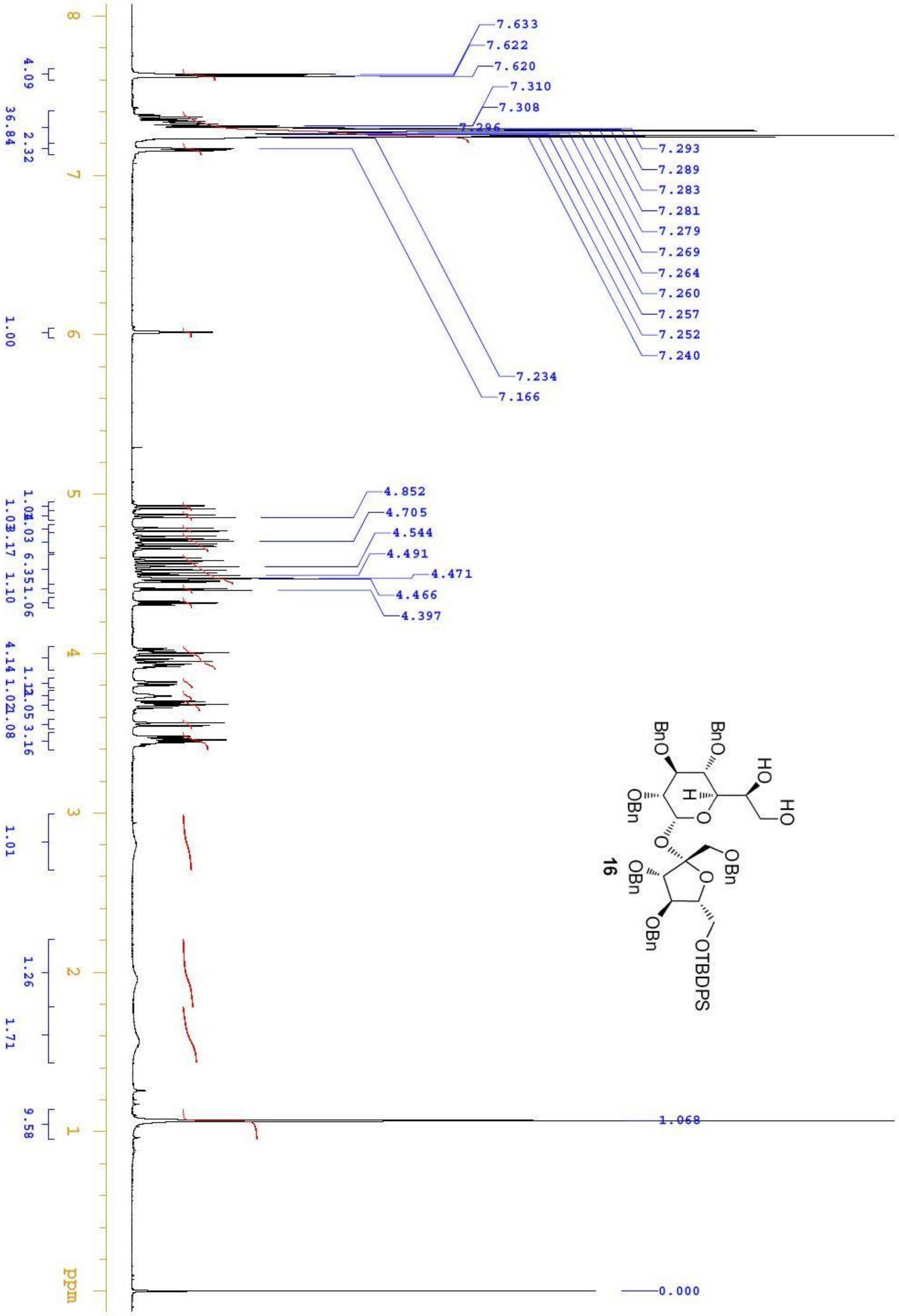
7.633
7.622
7.620
7.310
7.308
7.296
7.293
7.289
7.283
7.281
7.279
7.269
7.264
7.260
7.257
7.252
7.240
7.234
7.166
4.852
4.705
4.544
4.491
4.471
4.466
4.397
1.068
0.000
BnO
BnO
HO
HO
H
O
OBn
O
OBn
OBn
OBn
OTBDPS
16
ppm

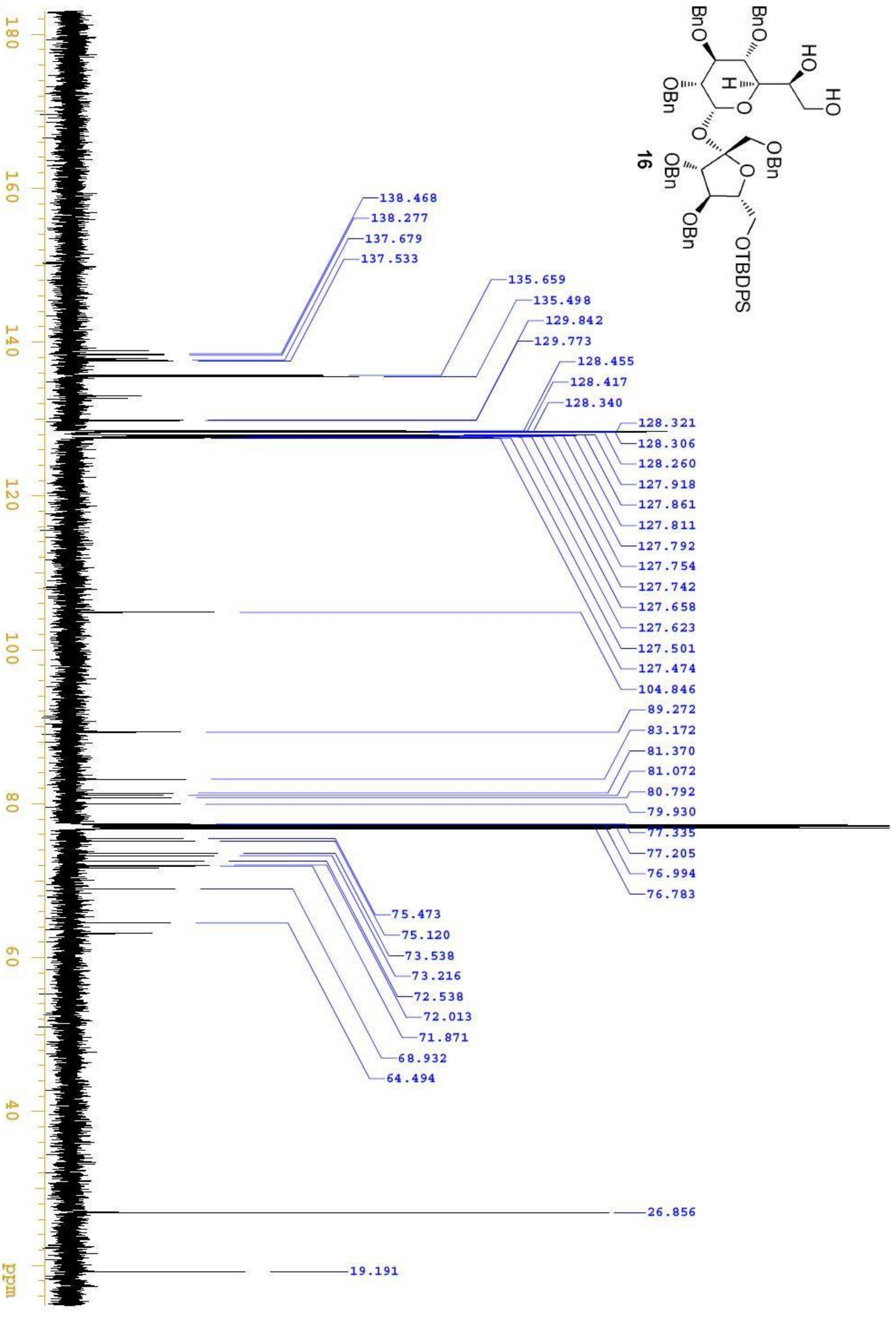

BnO
BnO
HO
HO
H
O
OBn
O
O
OBn
OBn
OBn
OTBDPS
16
138.468
138.277
137.679
137.533
135.659
135.498
129.842
129.773
128.455
128.417
128.340
128.321
128.306
128.260
127.918
127.861
127.811
127.792
127.754
127.742
127.658
127.623
127.501
127.474
104.846
89.272
83.172
81.370
81.072
80.792
79.930
77.335
77.205
76.994
76.783
75.473
75.120
73.538
73.216
72.538
72.013
71.871
68.932
64.494
26.856
19.191
180
160
140
120
100
80
60
40
ppm

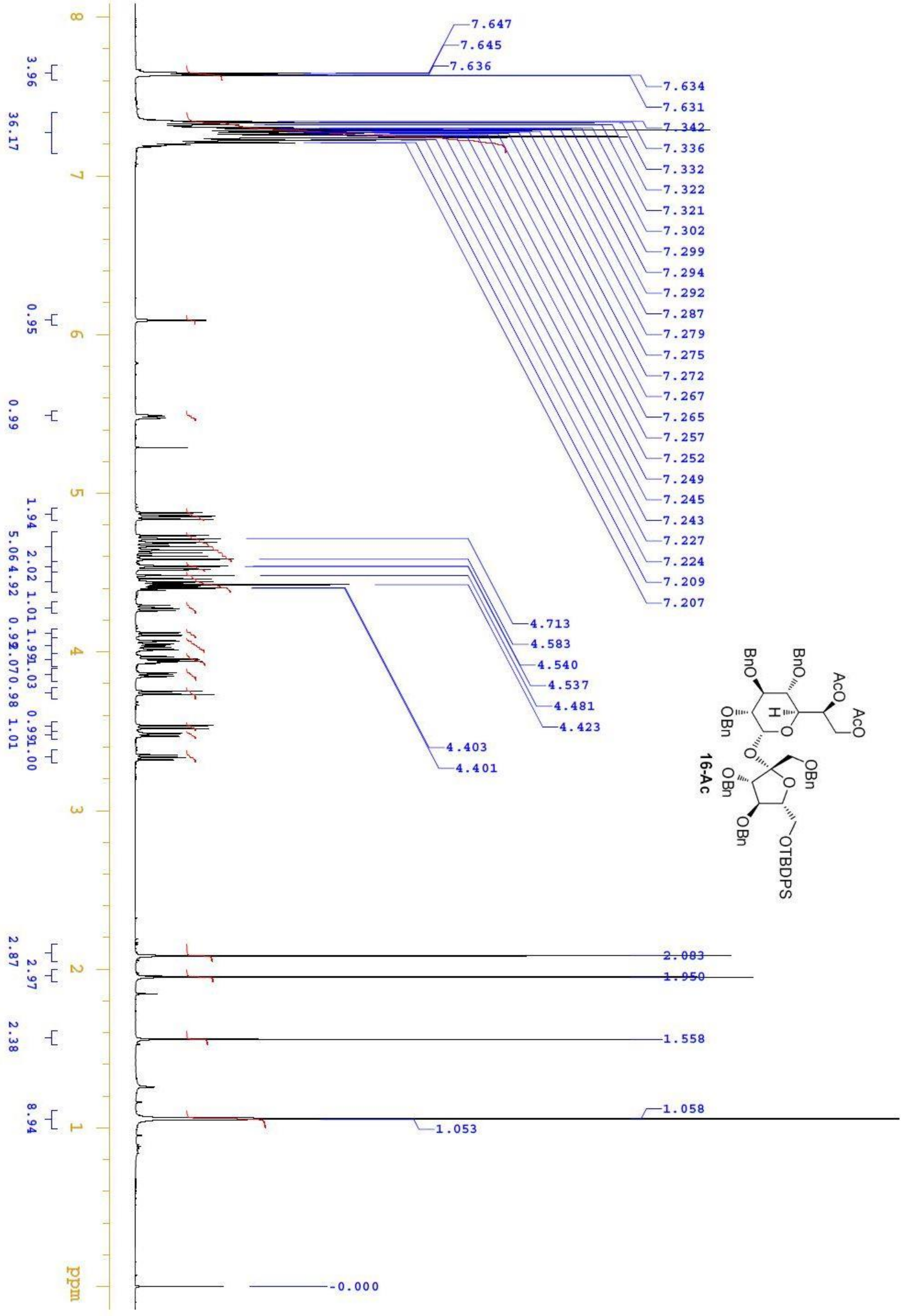
7.647
7.645
7.636
7.634
7.631
7.342
7.336
7.332
7.322
7.321
7.302
7.299
7.294
7.292
7.287
7.279
7.275
7.272
7.267
7.265
7.257
7.252
7.249
7.245
7.243
7.227
7.224
7.209
7.207
4.713
4.583
4.540
4.537
4.481
4.423
4.403
4.401
2.083
1.950
1.558
1.058
1.053
-0.000
3.96
36.17
0.95
0.99
1.94
5.06
4.92
2.02
1.01
2.87
2.97
2.38
8.94
ppm
BnO
AcO
OBn
OTBDPS
16-Ac

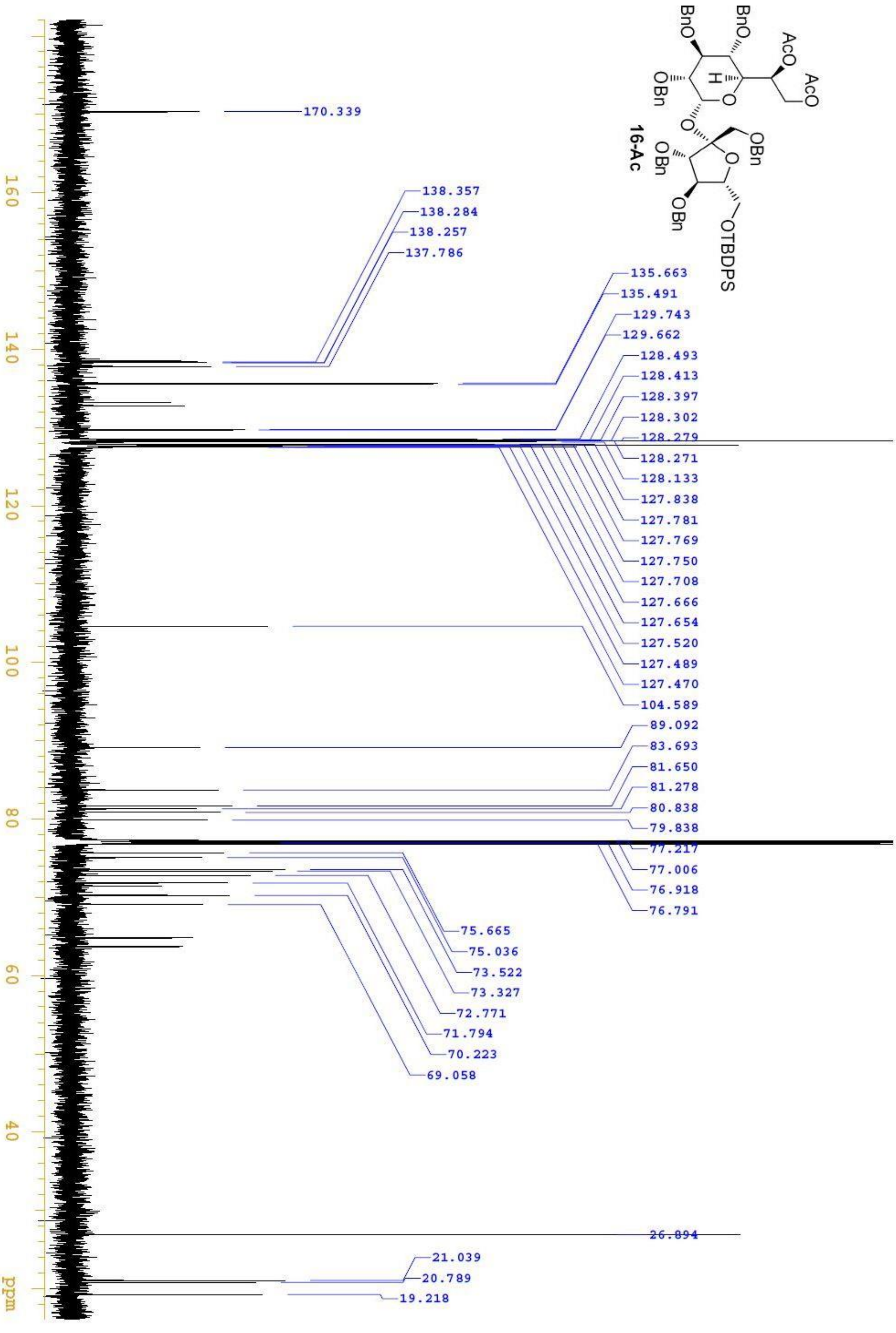
16-Ac
OTBDPS
170.339
138.357
138.284
138.257
137.786
135.663
135.491
129.743
129.662
128.493
128.413
128.397
128.302
128.279
128.271
128.133
127.838
127.781
127.769
127.750
127.708
127.666
127.654
127.520
127.489
127.470
104.589
89.092
83.693
81.650
81.278
80.838
79.838
77.217
77.006
76.918
76.791
75.665
75.036
73.522
73.327
72.771
71.794
70.223
69.058
26.894
21.039
20.789
19.218
160
140
120
100
80
60
40
ppm
